# Supplementary material for: Functional connectivity and GABAergic signaling modulate the enhancement effect of neurostimulation on mathematical learning
Source: PLoS Biol. 2025 Jul 1;23(7):e3003200. doi: 10.1371/journal.pbio.3003200 (PMC12212564; doi:10.1371/journal.pbio.3003200)
Supplement: S1 Text — (DOCX) [file pbio.3003200.s001.docx]

**S1 Text**. Additional Materials and Methods information.

*Participants*

The three stimulation groups (i.e., sham tRNS vs. active dlPFC-tRNS vs. active PPC-tRNS) did not differ in age (F(2,69)=.202, P=.82). All individuals met the safety criteria for tRNS and MRI, were medication-free, and had no history of psychiatric or neurological disease. Participants were compensated for their time and effort with £7.50 per hour of behavioral testing and scanning, and with £10 per hour for the tRNS sessions.

*Learning task*

In drill blocks, participants were presented with a given trial (e.g., “4 ♯ 12 = 17”), which could not be calculated as the required algorithm was not provided, but had to be memorized by rote learning. The participants were then presented with “4 ♯ 12 =” only and had to key in the correct answer (e.g., 17) based on the trial they remembered from earlier presentations. In calculation blocks, participants had to mentally insert a given set of two numbers (e.g., “19 § 4”) into an algorithm (e.g., “[first number – second number] + 1”, and enter the correct solution (in this case, 16). The task was presented on a computer screen and the task duration was approximately 30 minutes per day for five consecutive days. Three different sets of operations were combined with six (for drill learning) and 12 (for calculation learning) different sets of numbers (1) to balance task difficulty across the repeated sessions.

During the mathematical learning period, a card was placed on the table in front of the participants, reminding them of the algorithm that needed to be applied. Each learning session was accompanied by 20 minutes of either active or sham tRNS. As in previous studies (1-3), to keep the learning challenging and the participants engaged, the number of blocks, including 18 trials (3 repetitions of the 6 problems), increased across the first four learning days and then decreased slightly on the final learning day (first day: 10; second: 12; third: 14; fourth: 16, and fifth: 14) and, simultaneously, the duration of the arithmetic problem presentation decreased each day from 500ms to 250ms, to 125ms, to 62ms, and finally to 31ms on the last day.

The task started with a blank screen (200ms) that was followed by a fixation cross that was presented for 300ms. After an initial presentation of the problem, a mask (250ms) and blank screen (250ms) followed, and then the problem reappeared a second time until the participant had entered their response into the keypad, using a number from 0 to 9. In the calculation condition, there was unlimited time for the response, whereas in the drill condition problems had to be answered within a 3000ms interval. Feedback was presented for 500ms after every response, displaying either “Correct answer,” “Mistake,” or “No response” if the response came too late. In the latter two cases, the problem was presented repeatedly until answered correctly. Calculation and drill blocks were interleaved within each learning day and the order was counterbalanced. The main dependent variable, which we call learning, was defined as the mean reaction time (of correct responses) on each of the five days separately and for the two learning types separately (i.e., calculation and drill). The mean reaction time on a given day was defined by averaging the mean reaction time of the blocks that were completed on that day and, as mentioned above, the first day comprised 10 blocks (5 calculation blocks and 5 drill blocks), the second day 12, the third day 14, the fourth day 16, and the fifth day 14. We used reaction time as the dependent variable as it has previously been shown to be a sensitive measure for assessing skill acquisition (4), including under the present paradigm (3).

*tRNS*

The 5cmx5cm electrodes were covered with conductive synthetic rubber sponges soaked in saline, applied to the scalp surface, and held in place by rubber straps. In this between-participants design, participants were pseudo-randomly assigned to the three tRNS conditions (dlPFC-tRNS, PPC-tRNS, sham tRNS [half over the PPC and half over the dlPFC]) and matched according to age, gender, and baseline mathematical ability. The active tRNS conditions (i.e., dlPFC-tRNS and PPC-tRNS) involved an alternating high-frequency random current (hf-tRNS: 100-640Hz) at 1mA for 20 min. The sham tRNS condition featured a 15-second ramp-up and a separate 15-second ramp-down to either cortical site. This provided the initial percutaneous sensations experienced during stimulation and ensured the successful blinding of the participants (5). The participants and the experimenter who administered the learning tasks were blinded to the stimulation condition (i.e., active tRNS vs. sham tRNS). Fisher’s exact test confirmed adequate participant-blinding for active tRNS and sham tRNS (dlPFC-tRNS vs. sham tRNS, P=.33; PPC-tRNS vs. sham tRNS, P=.53; for further details, see **S11 Table**).

*MRS and MRS data analysis*

Outer volume suppression (OVS) was used to eliminate signal contamination from outside of the voxels. Shimming was performed using the GRE brain shim and the shim volume was centered around the voxel center and measured 3x3x3cm.

*MRS data analysis*

**Spectra Preprocessing:** Each scan was preprocessed in MATLAB using the FID-A toolkit (6). RF channels were combined using a weighted coil combination with the coil weights and phases determined by the baseline unsuppressed water scan. Retrospective frequency and phase drift correction were performed using time-domain spectral registration (7). The SPECIAL inversion-ON and inversion-OFF subspectra were subtracted to yield fully localized transients. Localized transients were aligned and averaged to yield the final spectra.

**LCmodel quantification**: Spectral quantification was performed in the LCmodel with in-house-generated basis sets. All metabolite basis functions were simulated in FID-A using the same RF pulse waveforms and pulse sequence timings as used experimentally. A basis function for macromolecule (MM) resonances was also included as a single MM basis function in the basis set. The MM basis function was determined by metabolite-nulled SPECIAL spectra acquired from an independent set of healthy volunteers. The corresponding water spectrum for each voxel acquisition was imported into the LCmodel as an internal reference. Metabolite concentrations were calculated in absolute units (mmol/L), including participant-specific corrections for fractions of grey matter (GM), white matter (WM), and cerebrospinal fluid (CSF) within the voxel(s) of interest (VOI).

**Tissue segmentation in each VOI and partial volume correction** (8, 9). The metabolite concentrations calculated in absolute molar units were then scaled using the selected regions’ structural properties (10). Namely, we segmented the images into different tissue classes using SPM12 and the Gannet 3.0 toolbox (11) to estimate the GM, WM, and CSF content of each voxel. Next, we calculated the number of GM, WM, and CSF voxels (in the T1-weighted MPRAGE image) within the three regions of interest separately in the native space. Subsequently, we divided these numbers (GM, WM, and CSF for every region) by the total number of voxels in the MRS VOI to create the corresponding GM, WM, and CSF fraction values per participant and region. As a final computation step, we scaled the absolute neurochemical values to these structural fractions using the following LCmodel (10) computation as can be seen in the following formula:

$Tissue-corrected concentration=\left( \left( absolute concentration*\frac{ATTMET}{CONCH2O*ATTH2O} \right) \right)*\left( \frac{\left( \left( H2OWM*\exp\left( -\frac{TE}{T2WM} \right)*\left( 1-\exp\left( -\frac{TR}{T1WM} \right) \right) \right)*WM fraction \right)+\left( \left( H2OGM*\exp\left( -\frac{TE}{T2GM} \right)*\left( 1-\exp\left( -\frac{TR}{T1GM} \right) \right) \right)*GM fraction \right)+\left( \left( H2OCSF*\exp\left( -\frac{TE}{T2CSF} \right)*\left( 1-\exp\left( -\frac{TR}{T1CSF} \right) \right) \right)*CSF fraction \right)}{1*\left( WM fraction+GM fraction \right)} \right)$

ATTMET is the attenuation of metabolite, and it was set to 1. ATTH2O is the attenuation of water due to relaxation and other effects and was set to 0.7 at TR of 4000ms, and TE of 8.5ms. The parameter CONCH2O was set to 35880 mmol/L (millimolars per liter) and referred to the tissue water concentration. The water concentration within the white matter (H2OWM), grey matter (H2OGM), and CSF (H2OCSF) were set to 36100, 43300, and 53800 mmol/L, respectively. The T2 relaxation of water in the white matter (T2WM), grey matter (T2GM), and CSF (T2CSF) were set to 79.2, 110, and 503ms, respectively. The T1 relaxation of water in the white matter (T1WM), grey matter (T1GM), and CSF (T1CSF) were set to 832, 1331, and 3817ms, respectively.

In terms of MRS exclusion criteria, we excluded (i) cases where the LCmodel-reported linewidth was >8 Hz, (ii) cases with a signal-to-noise ratio (SNR) below three standard deviations per time point per region, (iii) cases where GABA or glutamate concentration was not quantified in the LCmodel (i.e., the concentration was 0 and the metabolite-specific spectra were absent in the LCmodel output), and after these three steps we excluded any remaining cases (iv) where the metabolite concentration was beyond three standard deviations per metabolite (GABA or glutamate) per time point per region. For spectra plots and LCmodel fit estimates see **S5 Fig**. Please note that MRS alone cannot distinguish between the different pools of a given neurochemical, namely, MRS will detect the presence of a neurochemical regardless of the precise location which can be cellular, synaptic or extracellular. However, prior animal and human preclinical studies indicated that MRS-quantified levels of GABA and glutamate are a reasonable reflection of neurotransmitter levels and thus can be taken as a proxy for excitation/inhibition balance. Specifically, region-specific changes in GABA and glutamate assessed with MRS may reflect a shift in the balance of excitatory/inhibitory transmission in the context of preclinical studies including ageing, inflammation, and neurodegeneration neurotransmission (12).

*Resting fMRI*

Functional volumes were motion-corrected, slice time-corrected, segmented, normalized to a standardized (MNI) template, spatially smoothed with a Gaussian kernel (8mm FWHM), and pass-filtered (0.01Hz to Inf). We calculated the functional connectivity of the frontoparietal network using the seeds (see, **Fig 2A**) that are featured in the relevant network in CONN (13), namely, the right dlPFC (networks.FrontoParietal.RPFC: x=41, y=38, z=30), the left dlPFC (networks.FrontoParietal.LPFC: x=–43, y=33, z=28), the right PPC (networks.FrontoParietal.PPC: x=52, y=–52, z=45), and the left PPC (networks.FrontoParietal.PPC: x=–46, y=–58, z=49), to yield two frontoparietal functional connectivity scores for each participant: (i) the left frontoparietal connectivity (connection of left dlPFC with left PPC) and (ii) the right frontoparietal connectivity (connection of right dlPFC with right PPC). For the additional functional connectivity analyses (see below), we used the following occipital and hippocampal regions: atlas.Hippocampus r, atlas.Hippocampus l, and atlas.OP l (Occipital Pole Left), atlas.OP r (Occipital Pole Right), obtained from the standard atlas in CONN. We excluded cases where the functional connectivity measure was beyond three standard deviations per pair (e.g., dlPFC-PPC), per hemisphere, per time point.

*Statistical analyses*

The linear mixed effects analyses were performed in R (14) using *lme*, and the limits of the 95% confidence intervals were obtained using the package *intervals*. The tRNS condition (3 levels: dlPFC-tRNS, PPC-tRNS, and sham tRNS) and learning type (2 levels: calculation, drill) were the categorical fixed effects, and day, mathematical attainment, functional connectivity, first block drill reaction time, first block calculation reaction time, and neurochemical concentrations were the continuous fixed effects. In all these mixed effects analyses, we included random intercepts for participants and, when relevant, we applied the weight parameter for the stimulation condition. The term baseline (connectivity or attainment) refers to the measurement of said connectivity or attainment before the tRNS stimulation.

To assess whether learning was influenced by the baseline (pre-tRNS) dlPFC-PPC functional connectivity, we predicted learning (reaction time) using day, learning type (drill vs. calculation), and baseline frontoparietal connectivity, and we included random intercepts for participants.

Subsequently, we assessed the extent to which exciting specific frontoparietal pathways with active tRNS over one node (dlPFC) or another (PPC) can enhance learning. This was done by predicting learning (reaction time) using the tRNS condition (sham tRNS vs. dlPFC-tRNS vs. PPC-tRNS), day, learning type (drill vs. calculation), and baseline frontoparietal connectivity, and we included random intercepts for participants.

We subsequently predicted learning using the tRNS condition (sham tRNS vs. dlPFC-tRNS), day, learning type (drill vs. calculation), Δ neurochemical concentration (i.e., post-tRNS – baseline neurochemical concentration), and Δ frontoparietal connectivity.

**References**

1. T. Popescu *et al.*, Transcranial random noise stimulation mitigates increased difficulty in an arithmetic learning task. *Neuropsychologia* **81**, 255-264 (2016).

2. M. Delazer *et al.*, Learning by strategies and learning by drill—evidence from an fMRI study. *Neuroimage* **25**, 838-849 (2005).

3. A. Snowball *et al.*, Long-term enhancement of brain function and cognition using cognitive training and brain stimulation. *Current Biology* **23**, 987-992 (2013).

4. A. Newell, P. Rosenbloom, Mechanisms of skill acquisition. *Cognitive skills and their acquisition* (1981).

5. P. C. Gandiga, F. C. Hummel, L. G. Cohen, Transcranial DC stimulation (tDCS): a tool for double-blind sham-controlled clinical studies in brain stimulation. *Clinical neurophysiology* **117**, 845-850 (2006).

6. R. Simpson, G. A. Devenyi, P. Jezzard, T. J. Hennessy, J. Near, Advanced processing and simulation of MRS data using the FID appliance (FID‐A)—an open source, MATLAB‐based toolkit. *Magnetic resonance in medicine* **77**, 23-33 (2017).

7. J. Near *et al.*, Frequency and phase drift correction of magnetic resonance spectroscopy data by spectral registration in the time domain. *Magnetic resonance in medicine* **73**, 44-50 (2015).

8. J. Near *et al.*, Preprocessing, analysis and quantification in single‐voxel magnetic resonance spectroscopy: experts' consensus recommendations. *NMR in Biomedicine* **34**, e4257 (2021).

9. E. Dhamala *et al.*, Validation of in vivo MRS measures of metabolite concentrations in the human brain. *NMR in Biomedicine* **32**, e4058 (2019).

10. S. W. Provencher, Automatic quantitation of localized in vivo 1H spectra with LCModel. *NMR Biomed* **14**, 260-264 (2001).

11. R. A. Edden, N. A. Puts, A. D. Harris, P. B. Barker, C. J. Evans, Gannet: A batch‐processing tool for the quantitative analysis of gamma‐aminobutyric acid–edited MR spectroscopy spectra. *Journal of Magnetic Resonance Imaging* **40**, 1445-1452 (2014).

12. M. Febo, T. C. Foster, Preclinical magnetic resonance imaging and spectroscopy studies of memory, aging, and cognitive decline. *Frontiers in aging neuroscience* **8**, 158 (2016).

13. S. Whitfield-Gabrieli, A. Nieto-Castanon, Conn: a functional connectivity toolbox for correlated and anticorrelated brain networks. *Brain Connect* **2**, 125-141 (2012).

14. R. Core, TEAM, 2017. R: A language and environment for statistical computing. R Foundation for Statistical Computing, Vienna, Austria. *Online:* [*https://www*](https://www)*. r-project. org* (2022).
