# Supplementary material for: Functional connectivity and GABAergic signaling modulate the enhancement effect of neurostimulation on mathematical learning
Source: PLoS Biol. 2025 Jul 1;23(7):e3003200. doi: 10.1371/journal.pbio.3003200 (PMC12212564; doi:10.1371/journal.pbio.3003200)
Supplement: S1 Table — (A–D) Additional statistical analyses examining the role of baseline dlPFC-hippocampus connectivity and the role of baseline PPC–hippocampus connectivity in predicting academic learning (in the sham tRNS condition). Based on previous work showing that cortico-hippocampal connectivity is involved in math learning [36], we assessed whether dlPFC–hippocampus (or PPC–hippocampus) connectivity would explain variance in calculation or drill. To assess this possibility, we initially examine whether any of these four measures (i.e., dlPFC–hippocampus and PPC–hippocampus*left and right hemisphere) could predict learning using the day, learning type (drill versus calculation), baseline connectivity, and we included random intercepts for participants. Statistics: Value=regression coefficient, SE=standard error, DF=degrees of freedom, T=T-value, P=p-value, CI_L=confidence interval lower bound, CI_U=confidence interval upper bound, the suffix “_L” indicates the left hemisphere and the suffix “_R” indicates the right hemisphere. (DOCX) [file pbio.3003200.s002.docx]

**S1 Table A-D.** Additional statistical analyses examining the role of baseline dlPFC-hippocampus connectivity and the role of baseline PPC-hippocampus connectivity in predicting academic learning (in the sham tRNS condition). Based on previous work showing that cortico-hippocampal connectivity is involved in math learning (1), we assessed whether dlPFC-hippocampus (or PPC-hippocampus) connectivity would explain variance in calculation or drill. To assess this possibility, we initially examined whether any of these four measures (i.e., dlPFC-hippocampus and PPC-hippocampus*left and right hemisphere) could predict learning using the day, learning type (drill vs. calculation), baseline connectivity, and we included random intercepts for participants. **Statistics:** Value=regression coefficient, SE=standard error, DF=degrees of freedom, T=T-value, P=p-value, CI_L=confidence interval lower bound, CI_U=confidence interval upper bound, the suffix “_L” indicates the left hemisphere and the suffix “_R” indicates the right hemisphere. Interactor predictors are denoted by the * symbol.

|  | **Value** | **SE** | **DF** | **T** | **P** | **CI_L** | **CI_U** |
| --- | --- | --- | --- | --- | --- | --- | --- |
| **S1 Table A. Connectivity measure: Right PPC-hippocampus** | | | | | | | |
| (Intercept) | 3106.02 | 167.99 | 210 | 18.49 | 0.00 | 2774.87 | 3437.17 |
| TypeDrill | –2335.73 | 179.96 | 210 | –12.98 | 0.00 | –2690.48 | –1980.97 |
| Day | –297.70 | 38.37 | 210 | –7.76 | 0.00 | –373.34 | –222.07 |
| PPC–hippocampus_R | 1144.89 | 1523.66 | 22 | 0.75 | 0.46 | –2015.00 | 4304.77 |
| TypeDrill*Day | 250.60 | 54.26 | 210 | 4.62 | 0.00 | 143.64 | 357.57 |
| TypeDrill*PPC-hippocampus_R | –916.76 | 1632.24 | 210 | –0.56 | 0.57 | –4134.44 | 2300.92 |
| Day*PPC-hippocampus_R | 14.54 | 348.00 | 210 | 0.04 | 0.97 | –671.47 | 700.55 |
| TypeDrill*Day*PPC-hippocampus_R | 52.96 | 492.14 | 210 | 0.11 | 0.91 | –917.21 | 1023.12 |
| **S1 Table B. Connectivity measure: Left PPC-hippocampus** | | | | | | | |
| (Intercept) | 3117.89 | 160.83 | 210 | 19.39 | 0.00 | 2800.83 | 3434.94 |
| TypeDrill | –2341.21 | 171.31 | 210 | –13.67 | 0.00 | –2678.91 | –2003.50 |
| Day | –297.48 | 36.52 | 210 | –8.14 | 0.00 | –369.48 | –225.48 |
| PPC-hippocampus_L | 1569.82 | 1796.69 | 22 | 0.87 | 0.39 | –2156.29 | 5295.93 |
| TypeDrill*Day | 254.65 | 51.65 | 210 | 4.93 | 0.00 | 152.83 | 356.47 |
| TypeDrill*PPC-hippocampus_L | –1438.14 | 1913.72 | 210 | –0.75 | 0.45 | –5210.70 | 2334.42 |
| Day*PPC-hippocampus_L | 16.68 | 408.01 | 210 | 0.04 | 0.97 | –787.63 | 821.00 |
| TypeDrill*Day*PPC-hippocampus_L | –84.86 | 577.01 | 210 | –0.15 | 0.88 | –1222.33 | 1052.61 |
| **S1 Table C. Connectivity measure: Right dlPFC-hippocampus** | | | | | | | |
| (Intercept) | 3211.58 | 167.77 | 210 | 19.14 | 0.00 | 2880.85 | 3542.30 |
| TypeDrill | –2423.32 | 178.69 | 210 | –13.56 | 0.00 | –2775.58 | –2071.07 |
| Day | –304.74 | 38.10 | 210 | –8.00 | 0.00 | –379.84 | –229.63 |
| dlPFC-hippocampus_R | 961.02 | 984.67 | 22 | 0.98 | 0.34 | –1081.06 | 3003.08 |
| TypeDrill*Day | 256.91 | 53.88 | 210 | 4.77 | 0.00 | 150.70 | 363.12 |
| TypeDrill*dlPFC-hippocampus_R | –819.76 | 1048.78 | 210 | –0.78 | 0.44 | –2887.24 | 1247.73 |
| Day*dlPFC-hippocampus_R | –124.55 | 223.60 | 210 | –0.56 | 0.58 | –565.34 | 316.24 |
| TypeDrill*Day*dlPFC-hippocampus_R | 67.77 | 316.22 | 210 | 0.21 | 0.83 | –555.60 | 691.14 |
| **S1 Table D. Connectivity measure: Left dlPFC-hippocampus** | | | | | | | |
| (Intercept) | 3302.71 | 160.31 | 210 | 20.60 | 0.00 | 2986.69 | 3618.74 |
| TypeDrill | –2527.10 | 169.51 | 210 | –14.91 | 0.00 | –2861.25 | –2192.95 |
| Day | –309.94 | 36.14 | 210 | –8.58 | 0.00 | –381.18 | –238.70 |
| dlPFC-hippocampus_L | 2649.69 | 988.05 | 22 | 2.68 | 0.01 | 600.59 | 4698.78 |
| TypeDrill*Day | 263.90 | 51.11 | 210 | 5.16 | 0.00 | 163.15 | 364.65 |
| TypeDrill*dlPFC-hippocampus_L | –2720.15 | 1044.72 | 210 | –2.60 | 0.01 | –4779.62 | –660.67 |
| Day*dlPFC-hippocampus_L | –226.71 | 222.73 | 210 | –1.02 | 0.31 | –665.80 | 212.37 |
| TypeDrill*Day*dlPFC-hippocampus_L | 196.74 | 314.99 | 210 | 0.62 | 0.53 | –424.22 | 817.70 |

**References**

1. S. Qin *et al.*, Hippocampal-neocortical functional reorganization underlies children's cognitive development. *Nature neuroscience* **17**, 1263-1269 (2014).
