# Supplementary figures and images for: Functional connectivity and GABAergic signaling modulate the enhancement effect of neurostimulation on mathematical learning
Source: PLoS Biol. 2025 Jul 1;23(7):e3003200. doi: 10.1371/journal.pbio.3003200 (PMC12212564; doi:10.1371/journal.pbio.3003200)

**S1 Fig**.1H-MRS voxel (2x2x2cm) positions for **A**) the PPC, and **B**) dlPFC are shown on coronal slices.


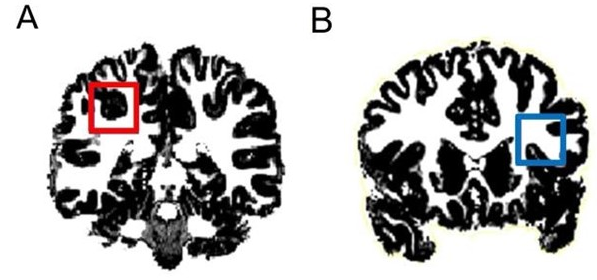

Supplement: S1 Fig — (DOCX) [file pbio.3003200.s003.docx]
