# Supplementary material for: Functional connectivity and GABAergic signaling modulate the enhancement effect of neurostimulation on mathematical learning
Source: PLoS Biol. 2025 Jul 1;23(7):e3003200. doi: 10.1371/journal.pbio.3003200 (PMC12212564; doi:10.1371/journal.pbio.3003200)

**S2 Fig.** Plotting the significant two-way interaction of left dlPFC-hippocampus connectivity with learning type from **S1 Table D**, where more positive connectivity was associated with poorer calculation learning, but no differences were observed for drill learning. The data underlying the results in **S2 Fig** can be found in **S4 Data**.


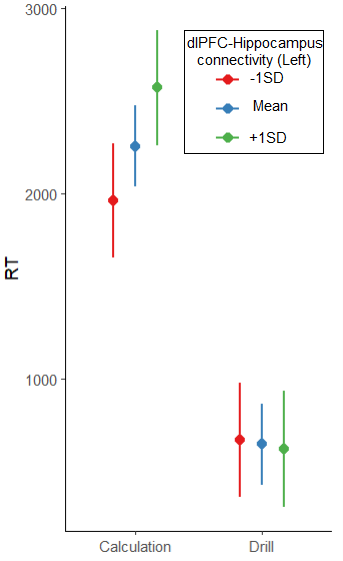

Supplement: S2 Fig — The data underlying the results in S2 Fig can be found in S4 Data. (DOCX) [file pbio.3003200.s004.docx]
