# Supplementary material for: Functional connectivity and GABAergic signaling modulate the enhancement effect of neurostimulation on mathematical learning
Source: PLoS Biol. 2025 Jul 1;23(7):e3003200. doi: 10.1371/journal.pbio.3003200 (PMC12212564; doi:10.1371/journal.pbio.3003200)
Supplement: S2 Table — Statistics: Value=regression coefficient, SE=standard error, DF=degrees of freedom, T=t-value, P=p-value, CI_L=confidence interval lower bound, CI_U= confidence interval upper bound, the suffix “_L” indicates the left hemisphere. Interactor predictors are denoted by the * symbol. (DOCX) [file pbio.3003200.s005.docx]

**S2 Table.** Additional statistical analyses examining the role of baseline frontoparietal connectivity after controlling for baseline dlPFC-hippocampus connectivity in predicting academic learning. **Statistics:** Value=regression coefficient, SE=standard error, DF=degrees of freedom, T=t-value, P=p-value, CI_L=confidence interval lower bound, CI_U= confidence interval upper bound, the suffix “_L” indicates the left hemisphere. Interactor predictors are denoted by the * symbol.

|  | **Value** | **SE** | **DF** | **T** | **P** | **CI_L** | **CI_U** |
| --- | --- | --- | --- | --- | --- | --- | --- |
| (Intercept) | 4479.01 | 541.62 | 207 | 8.27 | 0.00 | 3411.21 | 5546.81 |
| Day | –623.09 | 120.07 | 207 | –5.19 | 0.00 | –859.81 | –386.36 |
| TypeDrill | –3800.59 | 563.20 | 207 | –6.75 | 0.00 | –4910.93 | –2690.25 |
| Frontoparietal_L | –1852.65 | 814.53 | 21 | –2.27 | 0.03 | –3546.55 | –158.76 |
| dlPFC-hippocampus_L | 1949.96 | 1038.41 | 21 | 1.88 | 0.07 | –209.54 | 4109.46 |
| Day*TypeDrill | 614.82 | 169.81 | 207 | 3.62 | 0.00 | 280.04 | 949.60 |
| Day*Frontoparietal_L | 493.19 | 180.58 | 207 | 2.73 | 0.01 | 137.19 | 849.20 |
| TypeDrill*Frontoparietal_L | 2005.74 | 846.98 | 207 | 2.37 | 0.02 | 335.93 | 3675.54 |
| Day*dlPFC-hippocampus_L | –40.44 | 230.21 | 207 | –0.18 | 0.86 | –494.30 | 413.42 |
| TypeDrill*dlPFC-hippocampus_L | –1962.60 | 1079.79 | 207 | –1.82 | 0.07 | –4091.39 | 166.19 |
| Day*TypeDrill*Frontoparietal_L | –552.70 | 255.37 | 207 | –2.16 | 0.03 | –1056.16 | –49.23 |
| Day*TypeDrill*dlPFC-hippocampus_L | –12.01 | 325.57 | 207 | –0.04 | 0.97 | –653.86 | 629.85 |
