# Supplementary material for: Functional connectivity and GABAergic signaling modulate the enhancement effect of neurostimulation on mathematical learning
Source: PLoS Biol. 2025 Jul 1;23(7):e3003200. doi: 10.1371/journal.pbio.3003200 (PMC12212564; doi:10.1371/journal.pbio.3003200)

**S3 Fig**. Predicting learning (RT) in the drill learning task using left, Panel **A**, or right, Panel **B** frontoparietal connectivity across three levels of frontoparietal connectivity. Predicting RT in the drill learning task using left, Panel **C** or right, Panel **D** frontoparietal connectivity across three levels of right frontoparietal connectivity, –1SD (left panel), Mean (middle panel), and +1SD (right panel) and as a function of tRNS condition. The data underlying the results in **S3 Fig**  can be found in **S2 Data**.


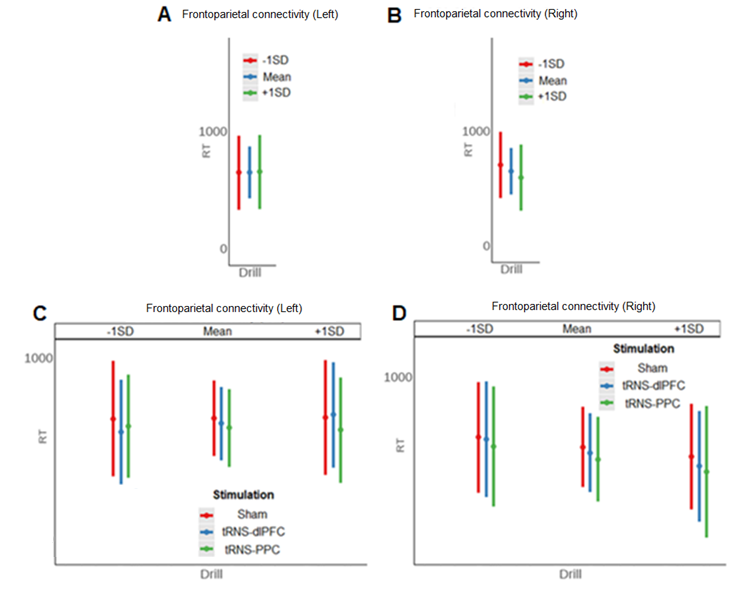

Supplement: S3 Fig — Predicting learning (RT) in the drill learning task using left, Panel A, or right, Panel B frontoparietal connectivity across three levels of frontoparietal connectivity. Predicting RT in the drill learning task using left, Panel C or right, Panel D frontoparietal connectivity across three levels of right frontoparietal connectivity, −1SD (left panel), Mean (middle panel), and +1SD (right panel) and as a function of tRNS condition. The data underlying the results in S3 Fig can be found in S2 Data. (DOCX) [file pbio.3003200.s006.docx]
