# Supplementary material for: Functional connectivity and GABAergic signaling modulate the enhancement effect of neurostimulation on mathematical learning
Source: PLoS Biol. 2025 Jul 1;23(7):e3003200. doi: 10.1371/journal.pbio.3003200 (PMC12212564; doi:10.1371/journal.pbio.3003200)
Supplement: S3 Table — To this end, we substituted frontoparietal connectivity with any of the four control connectivity measures: (i) left dlPFC–left occipital pole, (ii) right dlPFC–right occipital pole, (iii) left PPC–left occipital pole, and (iv) right PPC–right occipital pole. As can be seen in the tables below none of these four connectivity measures interacted with tRNS conditions and type to predict academic learning as the corresponding three-way interactions were not significant. Statistics: Value, regression coefficient; SE, standard error; DF, degrees of freedom, T, T-value; P, p-value. (DOCX) [file pbio.3003200.s007.docx]

**S3 Table.** To establish whether the functional connectivity results we observed are specific to frontoparietal connectivity rather than overall connectivity we assessed whether the control connectivity measures interacted with tRNS condition and type in predicting academic learning. To this end, we substituted frontoparietal connectivity with any of the four control connectivity measures: (i) left dlPFC-left occipital pole, (ii) right dlPFC-right occipital pole, (iii) left PPC-left occipital pole (iv) right PPC-right occipital pole. As can be seen in the tables below none of these four connectivity measures interacted with tRNS conditions and type to predict academic learning as the corresponding three-way interactions were not significant. **Statistics:** Value=regression coefficient, SE=standard error, DF=degrees of freedom, T=T-value, P=p-value.

|  | **Value** | **SE** | **DF** | **T** | **P** |  |
| --- | --- | --- | --- | --- | --- | --- |
| **Functional connectivity measure: Left dlPFC- Left Occipital Pole denoted here as FO_L** | | | | | |  |
| (Intercept) | 3024.5 | 221.1 | 612 | 13.7 | 0.0000 |  |
| TypeDrill | –2234.9 | 247.4 | 612 | –9.0 | 0.0000 |  |
| Day | –277.5 | 52.7 | 612 | –5.3 | 0.0000 |  |
| dlPFC-tRNS | –475.8 | 271.8 | 64 | –1.8 | 0.0849 |  |
| PPC-tRNS | –308.8 | 272.6 | 64 | –1.1 | 0.2616 |  |
| FO_L | 723.6 | 917.7 | 64 | 0.8 | 0.4333 |  |
| TypeDrill*Day | 224.3 | 74.6 | 612 | 3.0 | 0.0027 |  |
| TypeDrill*dlPFC-tRNS | 488.7 | 290.6 | 612 | 1.7 | 0.0931 |  |
| TypeDrill*PPC-tRNS | 141.6 | 301.7 | 612 | 0.5 | 0.6391 |  |
| Day*dlPFC-tRNS | 77.1 | 62.0 | 612 | 1.2 | 0.2139 |  |
| Day*PPC-tRNS | 44.5 | 64.3 | 612 | 0.7 | 0.4890 |  |
| TypeDrill*FO_L | –780.0 | 1027.0 | 612 | –0.8 | 0.4478 |  |
| Day*FO_L | –110.4 | 219.0 | 612 | –0.5 | 0.6143 |  |
| dlPFC-tRNS*FO_L | –259.6 | 1128.2 | 64 | –0.2 | 0.8187 |  |
| PPC-tRNS*FO_L | 3142.9 | 1362.7 | 64 | 2.3 | 0.0243 |  |
| TypeDrill*Day*dlPFC-tRNS | –82.1 | 87.6 | 612 | –0.9 | 0.3490 |  |
| TypeDrill*Day*PPC-tRNS | –17.7 | 91.0 | 612 | –0.2 | 0.8456 |  |
| TypeDrill*Day*FO_L | 160.4 | 309.6 | 612 | 0.5 | 0.6046 |  |
| TypeDrill*dlPFC-tRNS*FO_L | 210.8 | 1206.2 | 612 | 0.2 | 0.8614 |  |
| TypeDrill*PPC-tRNS*FO_L | –1633.4 | 1497.4 | 612 | –1.1 | 0.2758 |  |
| Day*dlPFC-tRNS*FO_L | 16.8 | 257.2 | 612 | 0.1 | 0.9478 |  |
| Day*PPC-tRNS*FO_L | –409.2 | 319.2 | 612 | –1.3 | 0.2004 |  |
| TypeDrill*Day*dlPFC-tRNS*FO_L | –50.8 | 363.7 | 612 | –0.1 | 0.8890 |  |
| TypeDrill*Day*PPC-tRNS*FO_L | 163.5 | 451.5 | 612 | 0.4 | 0.7174 |  |
| **Functional connectivity measure: Right dlPFC- Right Occipital Pole denoted here as FO_R** | | | | | |  |
| (Intercept) | 2872.1 | 182.2 | 612 | 15.8 | 0.0000 |  |
| TypeDrill | –2103.9 | 200.7 | 612 | –10.5 | 0.0000 |  |
| Day | –268.8 | 42.8 | 612 | –6.3 | 0.0000 |  |
| dlPFC-tRNS | –88.8 | 258.3 | 64 | –0.3 | 0.7322 |  |
| PPC-tRNS | –97.7 | 252.7 | 64 | –0.4 | 0.7004 |  |
| FO_R | 1648.5 | 632.6 | 64 | 2.6 | 0.0114 |  |
| TypeDrill*Day | 226.9 | 60.5 | 612 | 3.7 | 0.0002 |  |
| TypeDrill*dlPFC-tRNS | 207.1 | 266.9 | 612 | 0.8 | 0.4380 |  |
| TypeDrill*PPC-tRNS | –66.5 | 277.2 | 612 | –0.2 | 0.8106 |  |
| Day*dlPFC-tRNS | 33.5 | 56.9 | 612 | 0.6 | 0.5563 |  |
| Day*PPC-tRNS | 18.4 | 59.1 | 612 | 0.3 | 0.7558 |  |
| TypeDrill*FO_R | –1581.8 | 697.0 | 612 | –2.3 | 0.0236 |  |
| Day*FO_R | –166.2 | 148.6 | 612 | –1.1 | 0.2638 |  |
| dlPFC-tRNS*FO_R | –2799.7 | 1119.0 | 64 | –2.5 | 0.0149 |  |
| PPC-tRNS*FO_R | –88.1 | 1045.1 | 64 | –0.1 | 0.9331 |  |
| TypeDrill*Day*dlPFC-tRNS | –63.1 | 80.5 | 612 | –0.8 | 0.4328 |  |
| TypeDrill*Day*PPC-tRNS | 1.2 | 83.6 | 612 | 0.0 | 0.9885 |  |
| TypeDrill*Day*FO_R | 152.0 | 210.2 | 612 | 0.7 | 0.4697 |  |
| TypeDrill*dlPFC-tRNS*FO_R | 2043.8 | 1127.7 | 612 | 1.8 | 0.0704 |  |
| TypeDrill*PPC-tRNS*FO_R | 989.8 | 1145.1 | 612 | 0.9 | 0.3877 |  |
| Day*dlPFC-tRNS*FO_R | 312.2 | 240.4 | 612 | 1.3 | 0.1946 |  |
| Day*PPC-tRNS*FO_R | 46.6 | 244.1 | 612 | 0.2 | 0.8487 |  |
| TypeDrill*Day*dlPFC-tRNS*FO_R | –189.2 | 340.0 | 612 | –0.6 | 0.5780 |  |
| TypeDrill*Day*PPC-tRNS*FO_R | –178.0 | 345.3 | 612 | –0.5 | 0.6064 |  |
| **Functional connectivity measure: Left PPC- Left Occipital Pole denoted here as PO_L** | | | | | |  |
| (Intercept) | 3211.3 | 165.7 | 612 | 19.4 | 0.0000 |  |
| TypeDrill | –2434.8 | 185.5 | 612 | –13.1 | 0.0000 |  |
| Day | –305.3 | 39.5 | 612 | –7.7 | 0.0000 |  |
| dlPFC-tRNS | –482.5 | 210.0 | 64 | –2.3 | 0.0249 |  |
| PPC-tRNS | –263.6 | 222.7 | 64 | –1.2 | 0.2410 |  |
| PO_L | –707.4 | 864.0 | 64 | –0.8 | 0.4159 |  |
| TypeDrill*Day | 260.0 | 55.9 | 612 | 4.6 | 0.0000 |  |
| TypeDrill*dlPFC-tRNS | 546.1 | 222.4 | 612 | 2.5 | 0.0144 |  |
| TypeDrill*PPC-tRNS | 195.5 | 246.0 | 612 | 0.8 | 0.4270 |  |
| Day*dlPFC-tRNS | 71.6 | 47.4 | 612 | 1.5 | 0.1314 |  |
| Day*PPC-tRNS | 41.9 | 52.4 | 612 | 0.8 | 0.4251 |  |
| TypeDrill*PO_L | 744.8 | 967.2 | 612 | 0.8 | 0.4416 |  |
| Day*PO_L | 98.9 | 206.2 | 612 | 0.5 | 0.6317 |  |
| dlPFC-tRNS*PO_L | –969.7 | 1030.0 | 64 | –0.9 | 0.3500 |  |
| PPC-tRNS*PO_L | 3573.4 | 1419.2 | 64 | 2.5 | 0.0143 |  |
| TypeDrill*Day*dlPFC-tRNS | –92.7 | 67.1 | 612 | –1.4 | 0.1673 |  |
| TypeDrill*Day*PPC-tRNS | –34.4 | 74.2 | 612 | –0.5 | 0.6430 |  |
| TypeDrill*Day*PO_L | –87.7 | 291.6 | 612 | –0.3 | 0.7636 |  |
| TypeDrill*dlPFC-tRNS*PO_L | 136.1 | 1104.6 | 612 | 0.1 | 0.9020 |  |
| TypeDrill*PPC-tRNS*PO_L | –2919.4 | 1558.5 | 612 | –1.9 | 0.0615 |  |
| Day*dlPFC-tRNS*PO_L | 192.6 | 235.5 | 612 | 0.8 | 0.4138 |  |
| Day*PPC-tRNS*PO_L | –227.6 | 332.3 | 612 | –0.7 | 0.4937 |  |
| TypeDrill*Day*dlPFC-tRNS*PO_L | –46.5 | 333.1 | 612 | –0.1 | 0.8891 |  |
| TypeDrill*Day*PPC-tRNS*PO_L | 183.6 | 469.9 | 612 | 0.4 | 0.6962 |  |
| **Functional connectivity measure: Right PPC- Right Occipital Pole denoted here as PO_R** | | | | | |  |
| (Intercept) | 3180.79 | 155.72 | 603 | 20.43 | 0.00 |  |
| Day | –289.29 | 37.40 | 603 | –7.74 | 0.00 |  |
| TypeDrill | –2407.73 | 175.40 | 603 | –13.73 | 0.00 |  |
| PO_R | 171.54 | 1038.27 | 63 | 0.17 | 0.87 |  |
| dlPFC-tRNS | –448.72 | 199.72 | 63 | –2.25 | 0.03 |  |
| PPC-tRNS | –194.43 | 216.46 | 63 | –0.90 | 0.37 |  |
| Day*TypeDrill | 246.98 | 52.89 | 603 | 4.67 | 0.00 |  |
| Day*PO_R | –378.55 | 249.34 | 603 | –1.52 | 0.13 |  |
| TypeDrill*PO_R | 43.09 | 1169.49 | 603 | 0.04 | 0.97 |  |
| Day*dlPFC-tRNS | 58.56 | 45.14 | 603 | 1.30 | 0.20 |  |
| Day*PPC-tRNS | 23.43 | 51.33 | 603 | 0.46 | 0.65 |  |
| TypeDrill*dlPFC-tRNS | 513.69 | 211.71 | 603 | 2.43 | 0.02 |  |
| TypeDrill*PPC-tRNS | 151.86 | 240.76 | 603 | 0.63 | 0.53 |  |
| PO_R*dlPFC-tRNS | –2857.73 | 1253.51 | 63 | –2.28 | 0.03 |  |
| PO_R*PPC-tRNS | 2409.78 | 1490.92 | 63 | 1.62 | 0.11 |  |
| Day*TypeDrill*PO_R | 308.20 | 352.61 | 603 | 0.87 | 0.38 |  |
| Day*TypeDrill*dlPFC-tRNS | –81.57 | 63.83 | 603 | –1.28 | 0.20 |  |
| Day*TypeDrill*PPC-tRNS | –22.29 | 72.59 | 603 | –0.31 | 0.76 |  |
| Day*PO_R*dlPFC-tRNS | 763.64 | 286.91 | 603 | 2.66 | 0.01 |  |
| Day*PO_R*PPC-tRNS | 225.14 | 353.26 | 603 | 0.64 | 0.52 |  |
| TypeDrill*PO_R*dlPFC-tRNS | 1451.62 | 1345.75 | 603 | 1.08 | 0.28 |  |
| TypeDrill*PO_R*PPC-tRNS | –1302.35 | 1656.92 | 603 | –0.79 | 0.43 |  |
| Day*TypeDrill*PO_R*dlPFC-tRNS | –472.84 | 405.76 | 603 | –1.17 | 0.24 |  |
| Day*TypeDrill*PO_R*PPC-tRNS | –344.48 | 499.58 | 603 | –0.69 | 0.49 |  |
