# Supplementary material for: Functional connectivity and GABAergic signaling modulate the enhancement effect of neurostimulation on mathematical learning
Source: PLoS Biol. 2025 Jul 1;23(7):e3003200. doi: 10.1371/journal.pbio.3003200 (PMC12212564; doi:10.1371/journal.pbio.3003200)
Supplement: S4 Table — Statistics: Value, regression coefficient; SE, standard error, DF, degrees of freedom; T, t-value; P; p-value; CI_L, confidence interval lower bound; CI_U, confidence interval upper bound. Interactor predictors are denoted by the * symbol. The displayed “0.0” values in the accuracy sections of the table are due to rounding. (DOCX) [file pbio.3003200.s008.docx]

**S4 Table.** A table depicting the statistical results of the linear mixed-effects model predicting learning based on learning type (drill, calculation), tRNS condition (sham tRNS which is the reference group here, dlPFC-tRNS, PPC-tRNS), day (**S4 Table A** for reaction time, same as Table 1, and **S4 Table C** for accuracy), and a similar model that additionally featured the three covariates, mathematical attainment, first block calculation reaction time, and first block drill reaction time (**S4 Table B** for reaction time and **S4 Table D** for accuracy). **Statistics:** Value=regression coefficient, SE=standard error, DF=degrees of freedom, T=t-value, P=p-value, CI_L=confidence interval lower bound, CI_U=confidence interval upper bound. Interactor predictors are denoted by the * symbol. The displayed “0.0” values in the accuracy sections of the table are due to rounding.

|  | **Value** | **SE** | **DF** | **T** | **P** | **CI_L** | **CI_U** |  |
| --- | --- | --- | --- | --- | --- | --- | --- | --- |
| **S4 Table A** | | | | | | | | |
| (Intercept) | 3152.7 | 150.5 | 639 | 21.0 | 0.0000 | 2857.3 | 3448.2 |  |
| TypeDrill | -2373.1 | 166.6 | 639 | -14.2 | 0.0000 | -2700.4 | -2045.9 |  |
| Day | -297.1 | 35.5 | 639 | -8.4 | 0.0000 | -366.9 | -227.3 |  |
| dlPFC-tRNS | -535.6 | 196.5 | 69 | -2.7 | 0.0081 | -927.7 | -143.6 |  |
| PPC-tRNS | -343.4 | 209.2 | 69 | -1.6 | 0.1053 | -760.8 | 74.0 |  |
| TypeDrill*Day | 252.8 | 50.2 | 639 | 5.0 | 0.0000 | 154.1 | 351.4 |  |
| TypeDrill*dlPFC-tRNS | 543.1 | 205.4 | 639 | 2.6 | 0.0084 | 139.6 | 946.5 |  |
| TypeDrill*PPC-tRNS | 260.3 | 229.2 | 639 | 1.1 | 0.2566 | -189.8 | 710.3 |  |
| Day*dlPFC-tRNS | 82.9 | 43.8 | 639 | 1.9 | 0.0590 | -3.1 | 168.9 |  |
| Day*PPC-tRNS | 51.9 | 48.9 | 639 | 1.1 | 0.2888 | -44.1 | 147.8 |  |
| TypeDrill*Day*dlPFC-tRNS | -94.4 | 61.9 | 639 | -1.5 | 0.1280 | -216.0 | 27.2 |  |
| TypeDrill*Day*PPC-tRNS | -45.8 | 69.1 | 639 | -0.7 | 0.5082 | -181.4 | 89.9 |  |
| **S4 Table B** | | | | | | | | |
| (Intercept) | 3401.8 | 414.5 | 639 | 8.2 | 0.0000 | 2587.8 | 4215.8 |  |
| TypeDrill | -2373.1 | 167.2 | 639 | -14.2 | 0.0000 | -2701.4 | -2044.8 |  |
| Day | -297.1 | 35.6 | 639 | -8.3 | 0.0000 | -367.1 | -227.1 |  |
| dlPFC-tRNS | -514.1 | 154.6 | 66 | -3.3 | 0.0014 | -822.9 | -205.4 |  |
| PPC-tRNS | -326.4 | 171.3 | 66 | -1.9 | 0.0610 | -668.3 | 15.6 |  |
| First block drill | 0.3 | 0.1 | 66 | 3.1 | 0.0031 | 0.1 | 0.4 |  |
| First block calculation | 0.2 | 0.0 | 66 | 10.1 | 0.0000 | 0.1 | 0.2 |  |
| Mathematical attainment | -9.8 | 2.9 | 66 | -3.3 | 0.0015 | -15.6 | -3.9 |  |
| TypeDrill*Day | 252.8 | 50.4 | 639 | 5.0 | 0.0000 | 153.8 | 351.8 |  |
| TypeDrill*dlPFC-tRNS | 543.1 | 205.9 | 639 | 2.6 | 0.0086 | 138.7 | 947.5 |  |
| TypeDrill*PPC-tRNS | 260.3 | 229.1 | 639 | 1.1 | 0.2565 | -189.7 | 710.2 |  |
| Day*dlPFC-tRNS | 82.9 | 43.9 | 639 | 1.9 | 0.0596 | -3.4 | 169.1 |  |
| Day*PPC-tRNS | 51.9 | 48.9 | 639 | 1.1 | 0.2887 | -44.1 | 147.8 |  |
| TypeDrill*Day*dlPFC-tRNS | -94.4 | 62.1 | 639 | -1.5 | 0.1289 | -216.3 | 27.5 |  |
| TypeDrill*Day*PPC-tRNS | -45.8 | 69.1 | 639 | -0.7 | 0.5081 | -181.4 | 89.9 |  |
| **S4 Table C** | | | | | | | | |
| (Intercept) | 0.9 | 0.0 | 639 | 88.4 | 0.0000 | 0.9 | 0.9 |  |
| TypeDrill | 0.1 | 0.0 | 639 | 5.7 | 0.0000 | 0.0 | 0.1 |  |
| Day | 0.0 | 0.0 | 639 | 0.7 | 0.4932 | 0.0 | 0.0 |  |
| dlPFC-tRNS | 0.0 | 0.0 | 69 | -0.1 | 0.9602 | 0.0 | 0.0 |  |
| PPC-tRNS | 0.0 | 0.0 | 69 | -0.1 | 0.9200 | 0.0 | 0.0 |  |
| TypeDrill*Day | 0.0 | 0.0 | 639 | -3.6 | 0.0003 | 0.0 | 0.0 |  |
| TypeDrill*dlPFC-tRNS | 0.0 | 0.0 | 639 | -1.1 | 0.2793 | 0.0 | 0.0 |  |
| TypeDrill*PPC-tRNS | 0.0 | 0.0 | 639 | -0.8 | 0.4479 | -0.1 | 0.0 |  |
| Day*dlPFC-tRNS | 0.0 | 0.0 | 639 | 1.2 | 0.2419 | 0.0 | 0.0 |  |
| Day*PPC-tRNS | 0.0 | 0.0 | 639 | -0.7 | 0.5022 | 0.0 | 0.0 |  |
| TypeDrill*Day*dlPFC-tRNS | 0.0 | 0.0 | 639 | 0.2 | 0.8218 | 0.0 | 0.0 |  |
| TypeDrill*Day*PPC-tRNS | 0.0 | 0.0 | 639 | 0.5 | 0.6278 | 0.0 | 0.0 |  |
| **S4 Table D** | | | | | | | | |
| (Intercept) | 0.8 | 0.1 | 639 | 12.9 | 0.0000 | 0.6 | 0.9 |  |
| TypeDrill | 0.1 | 0.0 | 639 | 5.7 | 0.0000 | 0.0 | 0.1 |  |
| Day | 0.0 | 0.0 | 639 | 0.7 | 0.4934 | 0.0 | 0.0 |  |
| dlPFC-tRNS | 0.0 | 0.0 | 66 | 0.0 | 0.9618 | 0.0 | 0.0 |  |
| PPC-tRNS | 0.0 | 0.0 | 66 | 0.4 | 0.6669 | 0.0 | 0.0 |  |
| First block drill | 0.0 | 0.0 | 66 | 0.6 | 0.5251 | 0.0 | 0.0 |  |
| First block calculation | 0.0 | 0.0 | 66 | -0.4 | 0.6895 | 0.0 | 0.0 |  |
| Mathematical attainment | 0.0 | 0.0 | 66 | 2.9 | 0.0045 | 0.0 | 0.0 |  |
| TypeDrill*Day | 0.0 | 0.0 | 639 | -3.6 | 0.0003 | 0.0 | 0.0 |  |
| TypeDrill*dlPFC-tRNS | 0.0 | 0.0 | 639 | -1.1 | 0.2794 | 0.0 | 0.0 |  |
| TypeDrill*PPC-tRNS | 0.0 | 0.0 | 639 | -0.8 | 0.4476 | -0.1 | 0.0 |  |
| Day*dlPFC-tRNS | 0.0 | 0.0 | 639 | 1.2 | 0.2421 | 0.0 | 0.0 |  |
| Day*PPC-tRNS | 0.0 | 0.0 | 639 | -0.7 | 0.5019 | 0.0 | 0.0 |  |
| TypeDrill*Day*dlPFC-tRNS | 0.0 | 0.0 | 639 | 0.2 | 0.8218 | 0.0 | 0.0 |  |
| TypeDrill*Day*PPC-tRNS | 0.0 | 0.0 | 639 | 0.5 | 0.6276 | 0.0 | 0.0 |  |
