# Supplementary material for: Functional connectivity and GABAergic signaling modulate the enhancement effect of neurostimulation on mathematical learning
Source: PLoS Biol. 2025 Jul 1;23(7):e3003200. doi: 10.1371/journal.pbio.3003200 (PMC12212564; doi:10.1371/journal.pbio.3003200)
Supplement: S5 Table — Statistics: T, T-value; DF, degrees of freedom, P, p-value; CI_L, confidence interval lower bound; CI_U, confidence interval upper bound. (DOCX) [file pbio.3003200.s009.docx]

**S5 Table.** dlPFC-tRNS or PPC-tRNS did not significantly alter the levels of GABA and glutamate as shown by the independent sample t-test results. **Statistics:** T=T-value DF=degrees of freedom, , P=p-value, CI_L=confidence interval lower bound, CI_U=confidence interval upper bound.

| **Outcome** | **T** | **df** | **P** | **CI_L** | **CI_U** |
| --- | --- | --- | --- | --- | --- |
| Δ dlPFC GABA | 0.34 | 15 | 0.74 | –0.32 | 0.44 |
| Δ dlPFC glutamate | –0.11 | 25 | 0.91 | –1.36 | 1.22 |
| Δ PPC GABA | –0.54 | 20 | 0.60 | –0.84 | 0.49 |
| Δ PPC glutamate | 0.38 | 26 | 0.71 | –1.16 | 1.68 |
