# Supplementary material for: Functional connectivity and GABAergic signaling modulate the enhancement effect of neurostimulation on mathematical learning
Source: PLoS Biol. 2025 Jul 1;23(7):e3003200. doi: 10.1371/journal.pbio.3003200 (PMC12212564; doi:10.1371/journal.pbio.3003200)
Supplement: S6 Table — Statistics: Value, regression coefficient; SE, standard error; DF, degrees of freedom; T, t-value; P, p-value. Interactor predictors are denoted by the * symbol. Neurochemicals: dlPFC, dorsolateral prefrontal cortex; GABA, gamma-aminobutyric acid. A “Δ” prefix denotes a post-tRNS minus pre-tRNS difference score in that neurochemical or functional connectivity measure. (DOCX) [file pbio.3003200.s010.docx]

**S6 Table.** A table depicting the statistical results of the linear mixed-effects model predicting learning based on learning type (drill, calculation), tRNS condition (sham tRNS which is the reference group here, dlPFC-tRNS), dlPFC GABA concentration, day, and right frontoparietal connectivity (denoted as “FC”). **Statistics:** Value=regression coefficient, SE=standard error, DF=degrees of freedom, T=t-value, P=p-value. Interactor predictors are denoted by the * symbol. **Neurochemicals:** dlPFC=dorsolateral prefrontal cortex, GABA=gamma-aminobutyric acid. A “Δ” prefix denotes a post-tRNS minus pre-tRNS difference score in that neurochemical or functional connectivity measure.

|  | **Value** | **SE** | **DF** | **T** | **P** | **CI_L** | **CI_U** |
| --- | --- | --- | --- | --- | --- | --- | --- |
| **Functional connectivity measure: Right dlPFC- Right PPC obtained after vs. before the tRNS** | | | | | | | |
| (Intercept) | 2283.98 | 341.25 | 120 | 6.69 | 0.000 | 1608.33 | 2959.63 |
| Day | –277.35 | 79.71 | 120 | –3.48 | 0.001 | –435.17 | –119.54 |
| TypeDrill | –1504.91 | 373.86 | 120 | –4.03 | 0.000 | –2245.13 | –764.70 |
| Δ FC | 1101.76 | 738.08 | 8 | 1.49 | 0.174 | –600.26 | 2803.77 |
| Δ dlPFC GABA | –3288.98 | 2121.56 | 8 | –1.55 | 0.160 | –8181.31 | 1603.36 |
| dlPFC-tRNS | 260.92 | 380.05 | 8 | 0.69 | 0.512 | –615.47 | 1137.30 |
| Day*TypeDrill | 218.63 | 112.72 | 120 | 1.94 | 0.055 | –4.55 | 441.81 |
| Day*Δ FC | –327.50 | 172.40 | 120 | –1.90 | 0.060 | –668.83 | 13.83 |
| TypeDrill*Δ FC | –1147.36 | 808.61 | 120 | –1.42 | 0.159 | –2748.36 | 453.63 |
| Day*Δ dlPFC GABA | 96.55 | 495.54 | 120 | 0.19 | 0.846 | –884.59 | 1077.69 |
| TypeDrill*Δ dlPFC GABA | 3135.24 | 2324.30 | 120 | 1.35 | 0.180 | –1466.72 | 7737.19 |
| Δ FC*Δ dlPFC GABA | 15961.09 | 5899.88 | 8 | 2.71 | 0.027 | 2355.94 | 29566.24 |
| Day*dlPFC-tRNS | 39.97 | 88.84 | 120 | 0.45 | 0.654 | –135.92 | 215.86 |
| TypeDrill*dlPFC-tRNS | –156.98 | 416.68 | 120 | –0.38 | 0.707 | –981.97 | 668.02 |
| Δ FC*dlPFC-tRNS | 331.80 | 946.72 | 8 | 0.35 | 0.735 | –1851.34 | 2514.94 |
| Δ dlPFC GABA*dlPFC-tRNS | –219.83 | 2252.22 | 8 | –0.10 | 0.925 | –5413.46 | 4973.81 |
| Day*TypeDrill*Δ FC | 293.90 | 243.81 | 120 | 1.21 | 0.230 | –188.82 | 776.62 |
| Day*TypeDrill*Δ dlPFC GABA | –69.28 | 700.80 | 120 | –0.10 | 0.921 | –1456.82 | 1318.26 |
| Day*Δ FC*Δ dlPFC GABA | –1540.59 | 1378.06 | 120 | –1.12 | 0.266 | –4269.05 | 1187.88 |
| TypeDrill*Δ FC*Δ dlPFC GABA | –14922.50 | 6463.68 | 120 | –2.31 | 0.023 | –27720.13 | –2124.87 |
| Day*TypeDrill*dlPFC-tRNS | –71.94 | 125.63 | 120 | –0.57 | 0.568 | –320.69 | 176.80 |
| Day*Δ FC*dlPFC-tRNS | 21.17 | 221.47 | 120 | 0.10 | 0.924 | –417.33 | 459.66 |
| TypeDrill*Δ FC*dlPFC-tRNS | 623.18 | 1038.79 | 120 | 0.60 | 0.550 | –1433.54 | 2679.91 |
| Day*Δ dlPFC GABA*dlPFC-tRNS | 497.41 | 526.29 | 120 | 0.95 | 0.347 | –544.61 | 1539.44 |
| TypeDrill*Δ dlPFC GABA*dlPFC-tRNS | 492.60 | 2468.54 | 120 | 0.20 | 0.842 | –4394.93 | 5380.13 |
| Δ FC*Δ dlPFC GABA*dlPFC-tRNS | –18135.13 | 6069.28 | 8 | –2.99 | 0.017 | –32130.91 | –4139.35 |
| Day*TypeDrill*Δ FC*Δ dlPFC GABA | 1722.96 | 1948.87 | 120 | 0.88 | 0.378 | –2135.67 | 5581.59 |
| Day*TypeDrill*Δ FC*dlPFC-tRNS | –140.60 | 313.21 | 120 | –0.45 | 0.654 | –760.73 | 479.52 |
| Day*TypeDrill*Δ dlPFC GABA*dlPFC-tRNS | –572.23 | 744.29 | 120 | –0.77 | 0.444 | –2045.87 | 901.42 |
| Day*Δ FC*Δ dlPFC GABA*dlPFC-tRNS | 2081.91 | 1417.93 | 120 | 1.47 | 0.145 | –725.50 | 4889.32 |
| TypeDrill*Δ FC*Δ dlPFC GABA*dlPFC-tRNS | 18109.67 | 6650.70 | 120 | 2.72 | 0.007 | 4941.75 | 31277.59 |
| Day*TypeDrill*Δ FC*Δ dlPFC GABA*dlPFC-tRNS | –2341.54 | 2005.26 | 120 | –1.17 | 0.245 | –6311.82 | 1628.74 |
