# Supplementary material for: Functional connectivity and GABAergic signaling modulate the enhancement effect of neurostimulation on mathematical learning
Source: PLoS Biol. 2025 Jul 1;23(7):e3003200. doi: 10.1371/journal.pbio.3003200 (PMC12212564; doi:10.1371/journal.pbio.3003200)
Supplement: S7 Table — To do this, we extracted the 95% CI from Fig 3 and compared the two subgroups (i.e., Δ frontoparietal connectivity +1SD versus Δ frontoparietal connectivity −1SD). The CI to P value conversion was obtained from the following three steps (A) calculate the standard error: SE = (Upper CI − Lower CI)/(2 × 1.96) (B) calculate the test statistic: z = Est/SE (C) calculate the P-value: P = exp(−0.717 × z − 0.416 × z2) and the outcome of each step is documented in the tables below. This formula works only for positive z, so if z was negative, the minus sign was removed [1]. Statistics: CI_L, confidence interval lower bound; CI_U, confidence interval upper bound. (DOCX) [file pbio.3003200.s011.docx]

**S7 Table.** Additional analyses that unpack the interplay of Δ frontoparietal connectivity level (denoted as Δ Conn in the table below) and Δ dlPFC GABA on calculation learning separately for the plasticity state (i.e, Plasticity (Δ dlPFC GABA –1SD) and the stability state (i.e., Δ dlPFC GABA +1SD), for the sham tRNS condition (**S7 Table A**) and the dlPFC-tRNS condition (**S7 Table B**). To do this we extracted the 95% CI from **Fig 3** and compared the two subgroups (i.e., Δ frontoparietal connectivity +1SD vs Δ frontoparietal connectivity –1SD). The CI to P value conversion was obtained from the following three steps (A) calculate the standard error: SE = (Upper CI – Lower CI)/(2×1.96) (B) calculate the test statistic: z = Est/SE (C) calculate the P value: P = exp(–0.717×z – 0.416×z^2^) and the outcome of each step is documented in the tables below. This formula works only for positive z, so if z was negative, the minus sign was removed (1). **Statistics:** CI_L=confidence interval lower bound, CI_U= confidence interval upper bound.

| **S7 Table A (Sham tRNS)** | | | | | | | |
| --- | --- | --- | --- | --- | --- | --- | --- |
|  | Plasticity (Δ dlPFC GABA –1SD ) | | |  | Stability (Δ dlPFC GABA +1SD ) | | |
|  | CI_L | CI_U | Estimate |  | CI_L | CI_U | Estimate |
| Δ Conn –1SD | 2540.13 | 7182.53 | 4861.33 | Δ Conn –1SD | –1676 | 1757.53 | 40.77 |
| Δ Conn +1SD | 874.96 | 2246.41 | 1560.68 | Δ Conn +1SD | 846.6 | 2054.35 | 1450.48 |
| Diff | –1665.17 | –4936.12 | –3300.65 | Diff | 2522.6 | 296.82 | 1409.71 |
| SE |  |  | 834.43 | SE |  |  | 567.80 |
| z |  |  | -3.96 | z |  |  | -2.48 |
| P |  |  | 0.0001 | P |  |  | 0.013 |

| **S7 Table B (dlPFC tRNS)** | | | | | | | |
| --- | --- | --- | --- | --- | --- | --- | --- |
|  | Plasticity (Δ dlPFC GABA –1SD ) | | |  | Stability (Δ dlPFC GABA +1SD ) | | |
|  | CI_L | CI_U | Estimate |  | CI_L | CI_U | Estimate |
| Δ Conn –1SD | 2054.56 | 2692.62 | 2373.59 | Δ Conn –1SD | 979.97 | 1725.87 | 1352.92 |
| Δ Conn +1SD | 2242.2 | 3470.41 | 2856.31 | Δ Conn +1SD | 1042.99 | 2171.3 | 1607.15 |
| Diff | 187.64 | 777.79 | 482.72 | Diff | 63.02 | 445.43 | 254.23 |
| SE |  |  | 150.55 | SE |  |  | 97.55 |
| z |  |  | 3.21 | z |  |  | 2.61 |
| P |  |  | 0.0014 | P |  |  | 0.009 |

**References**

1. D. G. Altman, J. M. Bland, How to obtain the P value from a confidence interval. *Bmj* **343** (2011).
