# Supplementary material for: Functional connectivity and GABAergic signaling modulate the enhancement effect of neurostimulation on mathematical learning
Source: PLoS Biol. 2025 Jul 1;23(7):e3003200. doi: 10.1371/journal.pbio.3003200 (PMC12212564; doi:10.1371/journal.pbio.3003200)

**S4 Fig.** Plotting the four-way interaction of learning type*tRNS condition*right frontoparietal connectivity across three levels of condition dlPFC GABA, –1SD (Panel **A**), Mean (Panel **B**), and +1SD (Panel **C**), for ease in interpretability here we merely display the drill learning. The data underlying the results in **S4 Fig**  can be found in **S3 Data**.


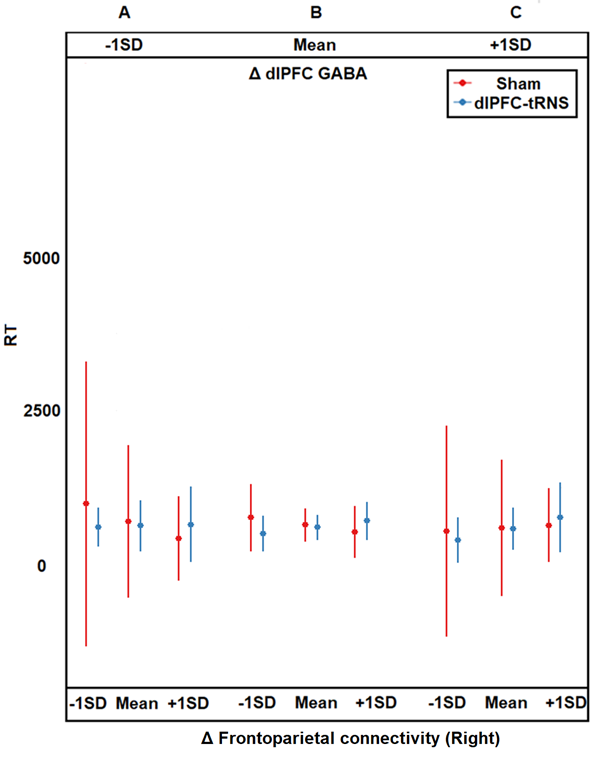

Supplement: S4 Fig — The data underlying the results in S4 Fig can be found in S3 Data. (DOCX) [file pbio.3003200.s012.docx]
