# Supplementary material for: Functional connectivity and GABAergic signaling modulate the enhancement effect of neurostimulation on mathematical learning
Source: PLoS Biol. 2025 Jul 1;23(7):e3003200. doi: 10.1371/journal.pbio.3003200 (PMC12212564; doi:10.1371/journal.pbio.3003200)
Supplement: S5 Fig — (A) The spectra from each region (dlPFC, PPC, and V1) and each time point (Pre, before tRNS; Post, after tRNS) separately. Spectra from all participants are overlaid. (B) The LCmodel fit estimates from each region (dlPFC, PPC, and V1) and each neurochemical (GABA = GABA, Glu = glutamate) separately before the tRNS. Fit estimates from all participants are overlaid. (C) The LCmodel fit estimates from each region (dlPFC, PPC, and V1) and each neurochemical (GABA = GABA, Glu = glutamate) separately after the tRNS. Fit estimates from all participants are overlaid. (DOCX) [file pbio.3003200.s013.docx]

**S5 Fig.** Spectra plots and LCmodel estimates.

**S5 Fig A.** The spectra from each region (dlPFC, PPC, and V1) and each time point (Pre=before tRNS, Post=after tRNS) separately. Spectra from all participants are overlaid.


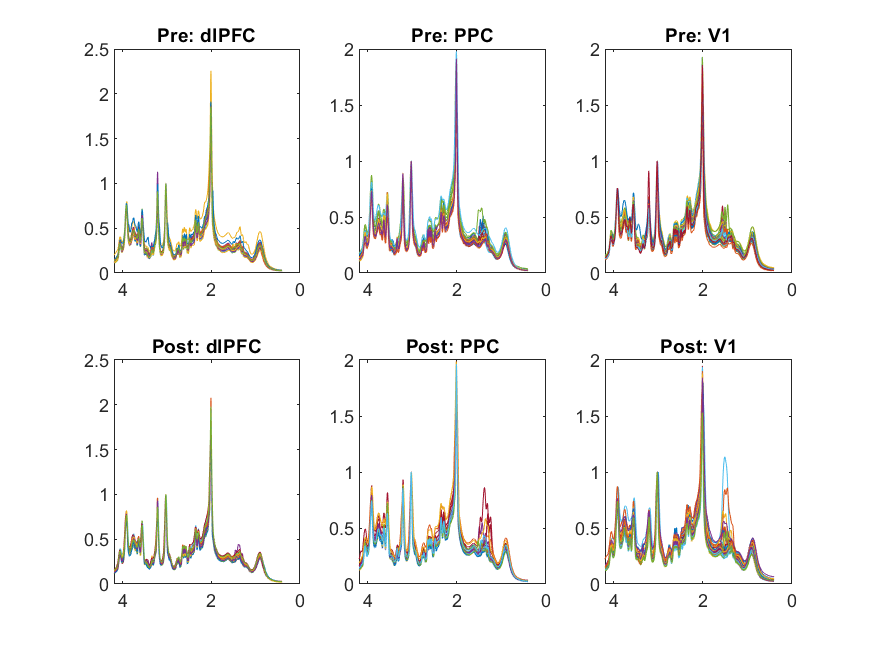


**S5 Fig B.** The LCmodel fit estimates from each region (dlPFC, PPC, and V1) and each neurochemical (GABA=GABA, Glu=glutamate) separately before the tRNS. Fit estimates from all participants are overlaid.

**
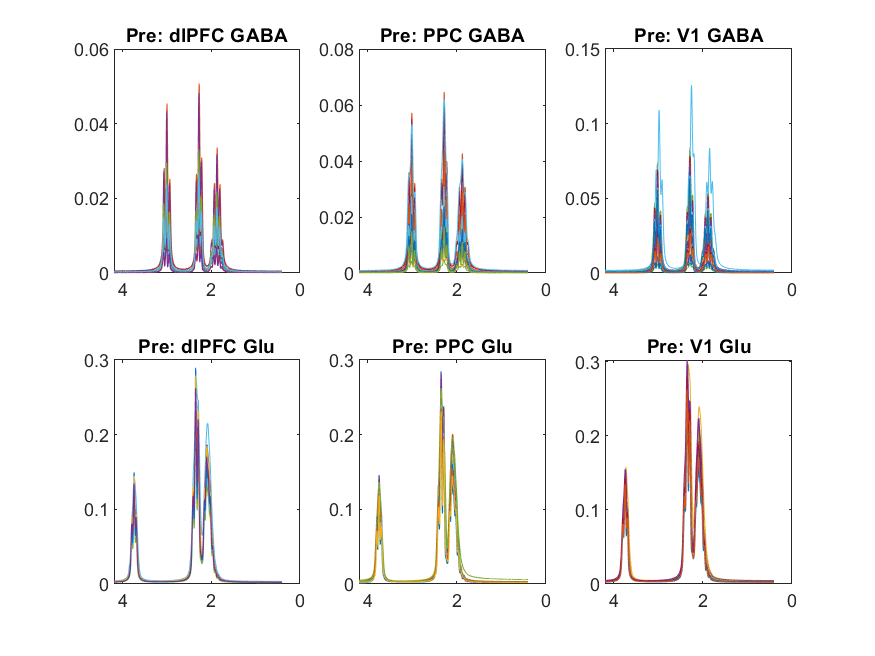
**

**S5 Fig C.** The LCmodel fit estimates from each region (dlPFC, PPC, and V1) and each neurochemical (GABA=GABA, Glu=glutamate) separately after the tRNS. Fit estimates from all participants are overlaid.

**
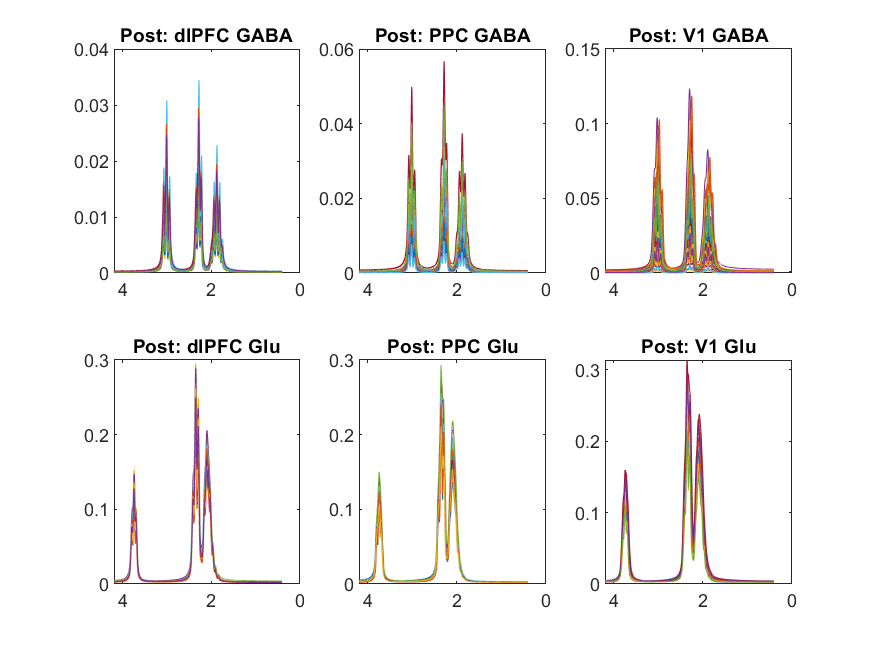
**
