# Supplementary material for: Functional connectivity and GABAergic signaling modulate the enhancement effect of neurostimulation on mathematical learning
Source: PLoS Biol. 2025 Jul 1;23(7):e3003200. doi: 10.1371/journal.pbio.3003200 (PMC12212564; doi:10.1371/journal.pbio.3003200)
Supplement: S8 Table — A “Δ” prefix denotes a post-tRNS minus pre-tRNS difference score in each cognitive measure. Statistics: T, t-value, DF, degrees of freedom; P, p-value; SE, standard error; CI_L, confidence interval lower bound; CI_U, confidence interval upper bound. (DOCX) [file pbio.3003200.s014.docx]

**S8 Table**. Independent sample t-test comparing the Sham tRNS vs dlPFC-tRNS (**S8 Table A**) or vs PPC-tRNS (**S8 Table B**) for the following scores: [Attention scores derived by the Attention Network Task (1) where Executive: ANT Executive network, Orienting: ANT Orienting network; Short term memory assessed by the Digit span task (2) and the Corsi Block tapping task (3) tests that included both forward and backward conditions]. A “Δ” prefix denotes a post-tRNS minus pre-tRNS difference score in each cognitive measure. **Statistics:** T=t-value, DF=degrees of freedom, P=p-value, SE=standard error, CI_L=confidence interval lower bound, CI_U= confidence interval upper bound.

|  | **T** | **DF** | **P** | **Mean** | **SE** | **CI_L** | **CI_U** |
| --- | --- | --- | --- | --- | --- | --- | --- |
| **S8 Table A** | | | | | | | |
| Δ Executive | 0.1 | 45 | 0.897 | 0.9 | 6.8 | -12.7 | 14.5 |
| Δ Orienting | -0.9 | 46 | 0.360 | -6.8 | 7.4 | -21.7 | 8.0 |
| Δ digitSpanFwd | -0.3 | 42 | 0.789 | -0.2 | 0.6 | -1.3 | 1.0 |
| Δ digitSpanBwd | 1.6 | 40 | 0.127 | 1.0 | 0.6 | -0.3 | 2.2 |
| Δ CorsiBwd | 0.5 | 39 | 0.593 | 0.3 | 0.5 | -0.8 | 1.4 |
| Δ CorsiFwd | -0.6 | 39 | 0.531 | -0.4 | 0.7 | -1.7 | 0.9 |
|  |  |  |  |  |  |  |  |
| **S8 Table B** | | | | | | | |
| Δ Executive | 0.0 | 45 | 0.971 | -0.2 | 6.4 | -13.2 | 12.7 |
| Δ Orienting | 0.7 | 46 | 0.476 | 5.1 | 7.1 | -9.1 | 19.3 |
| Δ digitSpanFwd | -1.5 | 41 | 0.135 | -1.0 | 0.6 | -2.2 | 0.3 |
| Δ digitSpanBwd | 0.6 | 39 | 0.576 | 0.4 | 0.6 | -0.9 | 1.7 |
| Δ CorsiBwd | 0.3 | 37 | 0.783 | 0.1 | 0.4 | -0.8 | 1.0 |
| Δ CorsiFwd | 0.3 | 38 | 0.767 | 0.2 | 0.6 | -1.1 | 1.4 |

**References**

1. J. Fan, B. D. McCandliss, T. Sommer, A. Raz, M. I. Posner, Testing the efficiency and independence of attentional networks. *Journal of cognitive neuroscience* **14**, 340-347 (2002).

2. D. Wechsler, *Wechsler adult intelligence scale-revised (WAIS-R)* (Psychological corporation, San Antonio, TX, 1981).

3. R. P. Kessels, M. J. Van Zandvoort, A. Postma, L. J. Kappelle, E. H. De Haan, The Corsi block-tapping task: standardization and normative data. *Applied neuropsychology* **7**, 252-258 (2000).
