# Supplementary material for: Functional connectivity and GABAergic signaling modulate the enhancement effect of neurostimulation on mathematical learning
Source: PLoS Biol. 2025 Jul 1;23(7):e3003200. doi: 10.1371/journal.pbio.3003200 (PMC12212564; doi:10.1371/journal.pbio.3003200)
Supplement: S9 Table — Statistics: Value, regression coefficient; SE, standard error; DF, degrees of freedom; T, T-value; P, p-value; CI_L, confidence interval lower bound; CI_U, confidence interval upper bound. Interactor predictors are denoted by the * symbol. (DOCX) [file pbio.3003200.s015.docx]

**S9 Table**. Additional statistical analyses examining the role of baseline frontoparietal connectivity in predicting accuracy. **Statistics:** Value= regression coefficient, SE=standard error, DF=degrees of freedom, T=T-value, P=p-value, CI_L=confidence interval lower bound, CI_U=confidence interval upper bound. Interactor predictors are denoted by the * symbol.

|  | **Value** | **SE** | **DF** | **T** | **P** | **CI_L** | **CI_U** |
| --- | --- | --- | --- | --- | --- | --- | --- |
| **S9 Table A (Right Hemisphere)** | | | | | | | |
| (Intercept) | 0.95 | 0.03 | 210 | 34.81 | 0.0000 | 0.89 | 1.00 |
| Day | 0.00 | 0.01 | 210 | 0.43 | 0.6679 | -0.01 | 0.02 |
| TypeDrill | 0.04 | 0.03 | 210 | 1.43 | 0.1547 | -0.02 | 0.10 |
| Frontoparietal_R | -0.04 | 0.04 | 22 | -0.98 | 0.3378 | -0.12 | 0.04 |
| Day*TypeDrill | -0.02 | 0.01 | 210 | -2.05 | 0.0419 | -0.04 | 0.00 |
| Day*Frontoparietal_R | 0.00 | 0.01 | 210 | -0.19 | 0.8514 | -0.02 | 0.02 |
| TypeDrill*Frontoparietal_R | 0.03 | 0.04 | 210 | 0.74 | 0.4595 | -0.05 | 0.11 |
| Day*TypeDrill*Frontoparietal_R | 0.01 | 0.01 | 210 | 0.76 | 0.4477 | -0.02 | 0.03 |
| **S9 Table B (Left Hemisphere)** | | | | | | | |
| (Intercept) | 0.90 | 0.03 | 210 | 25.63 | 0.0000 | 0.83 | 0.96 |
| Day | 0.00 | 0.01 | 210 | -0.09 | 0.9273 | -0.02 | 0.02 |
| TypeDrill | 0.11 | 0.04 | 210 | 2.72 | 0.0071 | 0.03 | 0.18 |
| Frontoparietal_L | 0.04 | 0.05 | 22 | 0.80 | 0.4337 | -0.07 | 0.15 |
| Day*TypeDrill | -0.02 | 0.01 | 210 | -1.92 | 0.0556 | -0.05 | 0.00 |
| Day*Frontoparietal_L | 0.00 | 0.01 | 210 | 0.30 | 0.7662 | -0.02 | 0.03 |
| TypeDrill*Frontoparietal_L | -0.07 | 0.06 | 210 | -1.16 | 0.2491 | -0.18 | 0.05 |
| Day*TypeDrill*Frontoparietal_L | 0.02 | 0.02 | 210 | 0.94 | 0.3462 | -0.02 | 0.05 |
