# Supplementary material for: Functional connectivity and GABAergic signaling modulate the enhancement effect of neurostimulation on mathematical learning
Source: PLoS Biol. 2025 Jul 1;23(7):e3003200. doi: 10.1371/journal.pbio.3003200 (PMC12212564; doi:10.1371/journal.pbio.3003200)
Supplement: S10 Table — Statistics: Value, regression coefficient; SE, standard error; DF, degrees of freedom; T, T-value; P, p-value. Interactor predictors are denoted by the * symbol. Neurochemicals: dlPFC, dorsolateral prefrontal cortex; GABA, gamma-aminobutyric acid. A “Δ” prefix denotes a post-tRNS minus pre-tRNS difference score in that neurochemical or functional connectivity measure. (DOCX) [file pbio.3003200.s016.docx]

**S10 Table.** A table depicting the statistical results of the linear mixed-effects model predicting accuracy based on learning type (drill, calculation), tRNS condition (sham tRNS which is the reference group here, dlPFC-tRNS), dlPFC GABA concentration, day, and right frontoparietal connectivity (denoted as “FC”). Statistics: Value=regression coefficient, SE=standard error, DF=degrees of freedom, T=T-value, P=p-value. Interactor predictors are denoted by the * symbol. Neurochemicals: dlPFC=dorsolateral prefrontal cortex, GABA=gamma-aminobutyric acid. A “Δ” prefix denotes a post-tRNS minus pre-tRNS difference score in that neurochemical or functional connectivity measure.

|  | **Value** | **SE** | **DF** | **T** | **P** | **CI_L** | **CI_U** |
| --- | --- | --- | --- | --- | --- | --- | --- |
| (Intercept) | 0.95 | 0.03 | 120 | 32.15 | 0.00 | 0.89 | 1.01 |
| Day | 0.00 | 0.01 | 120 | 0.27 | 0.79 | -0.01 | 0.02 |
| TypeDrill | 0.03 | 0.03 | 120 | 1.02 | 0.31 | -0.03 | 0.10 |
| Δ FC | -0.01 | 0.06 | 8 | -0.23 | 0.82 | -0.16 | 0.13 |
| Δ dlPFC GABA | 0.19 | 0.18 | 8 | 1.02 | 0.34 | -0.24 | 0.61 |
| dlPFC-tRNS | -0.02 | 0.03 | 8 | -0.53 | 0.61 | -0.09 | 0.06 |
| Day*TypeDrill | 0.00 | 0.01 | 120 | 0.05 | 0.96 | -0.02 | 0.02 |
| Day*Δ FC | 0.01 | 0.01 | 120 | 0.52 | 0.61 | -0.02 | 0.04 |
| TypeDrill*Δ FC | 0.06 | 0.07 | 120 | 0.86 | 0.39 | -0.08 | 0.20 |
| Day*Δ dlPFC GABA | -0.01 | 0.04 | 120 | -0.30 | 0.77 | -0.10 | 0.07 |
| TypeDrill*Δ dlPFC GABA | -0.21 | 0.20 | 120 | -1.05 | 0.29 | -0.61 | 0.19 |
| Δ FC*Δ dlPFC GABA | 0.13 | 0.51 | 8 | 0.25 | 0.81 | -1.05 | 1.30 |
| Day*dlPFC-tRNS | 0.00 | 0.01 | 120 | 0.53 | 0.60 | -0.01 | 0.02 |
| TypeDrill*dlPFC-tRNS | 0.03 | 0.04 | 120 | 0.79 | 0.43 | -0.04 | 0.10 |
| Δ FC*dlPFC-tRNS | -0.02 | 0.08 | 8 | -0.25 | 0.81 | -0.21 | 0.17 |
| Δ dlPFC GABA*dlPFC-tRNS | -0.17 | 0.19 | 8 | -0.88 | 0.40 | -0.62 | 0.28 |
| Day*TypeDrill*Δ FC | -0.03 | 0.02 | 120 | -1.20 | 0.23 | -0.07 | 0.02 |
| Day*TypeDrill*Δ dlPFC GABA | 0.06 | 0.06 | 120 | 0.95 | 0.34 | -0.06 | 0.18 |
| Day*Δ FC*Δ dlPFC GABA | -0.09 | 0.12 | 120 | -0.74 | 0.46 | -0.33 | 0.15 |
| TypeDrill*Δ FC*Δ dlPFC GABA | -0.05 | 0.56 | 120 | -0.09 | 0.93 | -1.17 | 1.06 |
| Day*TypeDrill*dlPFC-tRNS | -0.02 | 0.01 | 120 | -1.40 | 0.16 | -0.04 | 0.01 |
| Day*Δ FC*dlPFC-tRNS | -0.01 | 0.02 | 120 | -0.42 | 0.67 | -0.05 | 0.03 |
| TypeDrill*Δ FC*dlPFC-tRNS | -0.02 | 0.09 | 120 | -0.18 | 0.86 | -0.19 | 0.16 |
| Day*Δ dlPFC GABA*dlPFC-tRNS | 0.02 | 0.05 | 120 | 0.40 | 0.69 | -0.07 | 0.11 |
| TypeDrill*Δ dlPFC GABA*dlPFC-tRNS | 0.24 | 0.21 | 120 | 1.12 | 0.27 | -0.19 | 0.66 |
| Δ FC*Δ dlPFC GABA*dlPFC-tRNS | -0.36 | 0.52 | 8 | -0.68 | 0.52 | -1.56 | 0.85 |
| Day*TypeDrill*Δ FC*Δ dlPFC GABA | -0.02 | 0.17 | 120 | -0.09 | 0.93 | -0.35 | 0.32 |
| Day*TypeDrill*Δ FC*dlPFC-tRNS | 0.01 | 0.03 | 120 | 0.50 | 0.62 | -0.04 | 0.07 |
| Day*TypeDrill*Δ dlPFC GABA*dlPFC-tRNS | -0.08 | 0.06 | 120 | -1.18 | 0.24 | -0.20 | 0.05 |
| Day*Δ FC*Δ dlPFC GABA*dlPFC-tRNS | 0.11 | 0.12 | 120 | 0.86 | 0.39 | -0.14 | 0.35 |
| TypeDrill*Δ FC*Δ dlPFC GABA*dlPFC-tRNS | 0.28 | 0.58 | 120 | 0.48 | 0.63 | -0.87 | 1.42 |
| Day*TypeDrill*Δ FC*Δ dlPFC GABA*dlPFC-tRNS | -0.06 | 0.17 | 120 | -0.32 | 0.75 | -0.40 | 0.29 |
