# Supplementary material for: Functional connectivity and GABAergic signaling modulate the enhancement effect of neurostimulation on mathematical learning
Source: PLoS Biol. 2025 Jul 1;23(7):e3003200. doi: 10.1371/journal.pbio.3003200 (PMC12212564; doi:10.1371/journal.pbio.3003200)
Supplement: S11 Table — Cells contain the number of responses of each type. There was a similar response to condition (active tRNS vs. sham tRNS) in each group (Fisher’s exact test dlPFC-tRNS vs. sham tRNS, P = 0.33, PPC-tRNS vs. sham tRNS, P = 0.53). (DOCX) [file pbio.3003200.s017.docx]

**S11 Table**. Participants' guesses of the tRNS condition at the end of learning. Cells contain the number of responses of each type. There was a similar response to condition (active tRNS vs. sham tRNS) in each group (Fisher's exact test dlPFC-tRNS vs. sham tRNS, P=0.33, PPC-tRNS vs. sham tRNS, P=0.53).

| Stimulation/Response | "active tRNS" | "sham tRNS" | "I do not know" |
| --- | --- | --- | --- |
| dlPFC | 5 | 17 | 2 |
| PPC | 6 | 16 | 2 |
| sham tRNS | 8 | 13 | 3 |
