# Supplementary material for: Functional connectivity and GABAergic signaling modulate the enhancement effect of neurostimulation on mathematical learning
Source: PLoS Biol. 2025 Jul 1;23(7):e3003200. doi: 10.1371/journal.pbio.3003200 (PMC12212564; doi:10.1371/journal.pbio.3003200)
Supplement: S12 Table — (DOCX) [file pbio.3003200.s018.docx]

**S12 Table.** Mean and standard deviation (SD) of reaction time and accuracy separately for learning type (Drill, Calculation), stimulation groups (Sham-tRNS, dlPFC-tRNS and PPC-tRNS), day (1 to 5), and block.

|  | **Sham-tRNS** | | **dlPFC-tRNS** | | **PPC-tRNS** | |
| --- | --- | --- | --- | --- | --- | --- |
|  | **Mean** | **SD** | **Mean** | **SD** | **Mean** | **SD** |
| Drill RT Day 1 Block 1 | 902.67 | 304.85 | 973.53 | 404.03 | 810.95 | 385.84 |
| Drill RT Day 1 Block 2 | 809.32 | 266.04 | 788.87 | 279.58 | 745.60 | 370.08 |
| Drill RT Day 1 Block 3 | 710.17 | 224.93 | 718.21 | 277.99 | 676.39 | 389.44 |
| Drill RT Day 1 Block 4 | 722.44 | 250.27 | 665.72 | 270.95 | 676.35 | 378.87 |
| Drill RT Day 1 Block 5 | 675.91 | 254.74 | 646.54 | 249.55 | 727.29 | 356.28 |
| Drill RT Day 2 Block 1 | 820.03 | 328.62 | 735.56 | 336.07 | 689.60 | 335.65 |
| Drill RT Day 2 Block 2 | 721.15 | 301.38 | 711.63 | 356.67 | 621.06 | 276.61 |
| Drill RT Day 2 Block 3 | 700.75 | 284.57 | 656.41 | 328.73 | 576.77 | 258.47 |
| Drill RT Day 2 Block 4 | 686.87 | 281.11 | 661.63 | 298.31 | 549.99 | 257.44 |
| Drill RT Day 2 Block 5 | 632.59 | 256.60 | 667.80 | 334.35 | 547.00 | 273.86 |
| Drill RT Day 2 Block 6 | 629.17 | 231.41 | 680.85 | 353.71 | 529.44 | 205.73 |
| Drill RT Day 3 Block 1 | 670.32 | 281.09 | 648.72 | 286.20 | 590.00 | 235.38 |
| Drill RT Day 3 Block 2 | 647.71 | 267.86 | 586.72 | 248.28 | 522.13 | 213.04 |
| Drill RT Day 3 Block 3 | 592.92 | 230.55 | 564.22 | 258.47 | 546.99 | 249.57 |
| Drill RT Day 3 Block 4 | 591.18 | 226.84 | 549.74 | 231.43 | 503.68 | 206.98 |
| Drill RT Day 3 Block 5 | 598.57 | 276.38 | 535.64 | 254.15 | 474.42 | 183.37 |
| Drill RT Day 3 Block 6 | 589.53 | 232.97 | 550.96 | 270.75 | 487.82 | 213.51 |
| Drill RT Day 3 Block 7 | 547.02 | 225.18 | 537.07 | 212.53 | 481.00 | 194.76 |
| Drill RT Day 4 Block 1 | 650.57 | 215.47 | 577.43 | 187.06 | 560.27 | 187.56 |
| Drill RT Day 4 Block 2 | 590.89 | 207.31 | 556.96 | 205.12 | 501.91 | 199.02 |
| Drill RT Day 4 Block 3 | 544.46 | 186.24 | 532.98 | 175.92 | 514.28 | 196.40 |
| Drill RT Day 4 Block 4 | 530.11 | 210.41 | 519.51 | 175.90 | 492.47 | 200.65 |
| Drill RT Day 4 Block 5 | 514.04 | 199.87 | 513.14 | 189.01 | 497.25 | 216.28 |
| Drill RT Day 4 Block 6 | 521.69 | 204.92 | 522.13 | 214.94 | 512.74 | 250.85 |
| Drill RT Day 4 Block 7 | 505.95 | 196.25 | 491.85 | 174.61 | 480.55 | 199.07 |
| Drill RT Day 4 Block 8 | 516.63 | 203.32 | 496.37 | 177.21 | 468.76 | 187.46 |
| Drill RT Day 5 Block 1 | 728.08 | 208.88 | 623.92 | 213.22 | 665.82 | 235.39 |
| Drill RT Day 5 Block 2 | 642.13 | 201.47 | 577.26 | 205.38 | 603.79 | 208.60 |
| Drill RT Day 5 Block 3 | 625.39 | 218.69 | 562.43 | 237.26 | 566.54 | 236.11 |
| Drill RT Day 5 Block 4 | 613.26 | 196.43 | 562.42 | 202.54 | 567.47 | 242.13 |
| Drill RT Day 5 Block 5 | 583.75 | 208.46 | 546.47 | 210.24 | 533.75 | 223.58 |
| Drill RT Day 5 Block 6 | 570.95 | 198.55 | 512.45 | 199.28 | 564.06 | 240.49 |
| Drill RT Day 5 Block 7 | 563.78 | 213.43 | 526.97 | 183.17 | 539.38 | 242.65 |
| Calculation RT Day 1 Block 1 | 4102.23 | 1885.26 | 3785.48 | 1824.52 | 3773.01 | 1871.60 |
| Calculation RT Day 1 Block 2 | 3266.25 | 1530.21 | 2616.45 | 1178.83 | 2947.57 | 1583.46 |
| Calculation RT Day 1 Block 3 | 3058.15 | 1214.40 | 2477.21 | 1140.13 | 2805.17 | 1419.94 |
| Calculation RT Day 1 Block 4 | 2688.55 | 1196.16 | 2203.18 | 819.50 | 2347.05 | 1134.50 |
| Calculation RT Day 1 Block 5 | 2489.77 | 1199.64 | 2144.43 | 810.72 | 2297.03 | 1173.12 |
| Calculation RT Day 2 Block 1 | 2871.76 | 1379.71 | 2281.99 | 865.73 | 2600.58 | 1353.24 |
| Calculation RT Day 2 Block 2 | 2433.37 | 1096.62 | 2185.16 | 997.84 | 2094.46 | 1089.58 |
| Calculation RT Day 2 Block 3 | 2481.73 | 1185.74 | 2045.35 | 716.15 | 2093.59 | 1083.94 |
| Calculation RT Day 2 Block 4 | 2299.56 | 952.06 | 1947.41 | 679.16 | 2085.75 | 966.15 |
| Calculation RT Day 2 Block 5 | 2056.18 | 908.67 | 1896.43 | 769.86 | 1940.78 | 912.14 |
| Calculation RT Day 2 Block 6 | 1949.51 | 934.42 | 1869.15 | 632.62 | 1841.44 | 869.63 |
| Calculation RT Day 3 Block 1 | 2234.42 | 851.55 | 1995.87 | 751.50 | 1949.83 | 741.64 |
| Calculation RT Day 3 Block 2 | 2311.68 | 1053.28 | 1848.85 | 632.12 | 1863.39 | 742.69 |
| Calculation RT Day 3 Block 3 | 2116.92 | 949.46 | 1770.64 | 585.08 | 1832.37 | 814.48 |
| Calculation RT Day 3 Block 4 | 2092.09 | 1162.40 | 1744.52 | 566.24 | 1956.05 | 831.50 |
| Calculation RT Day 3 Block 5 | 1962.64 | 1114.22 | 1704.03 | 597.54 | 1878.57 | 828.80 |
| Calculation RT Day 3 Block 6 | 2019.76 | 1051.91 | 1688.22 | 611.40 | 1787.63 | 778.31 |
| Calculation RT Day 3 Block 7 | 1868.57 | 985.01 | 1618.73 | 620.71 | 1844.39 | 897.82 |
| Calculation RT Day 4 Block 1 | 2019.62 | 739.90 | 1671.43 | 515.80 | 1807.79 | 704.38 |
| Calculation RT Day 4 Block 2 | 1875.84 | 702.41 | 1588.18 | 480.57 | 1787.59 | 857.73 |
| Calculation RT Day 4 Block 3 | 1911.42 | 883.38 | 1637.59 | 504.20 | 1748.96 | 718.07 |
| Calculation RT Day 4 Block 4 | 1992.85 | 986.21 | 1782.84 | 780.31 | 1880.85 | 839.67 |
| Calculation RT Day 4 Block 5 | 1766.88 | 845.68 | 1709.75 | 690.57 | 1685.95 | 740.40 |
| Calculation RT Day 4 Block 6 | 1948.99 | 1009.91 | 1643.32 | 635.87 | 1882.42 | 946.75 |
| Calculation RT Day 4 Block 7 | 1804.06 | 843.55 | 1525.88 | 580.97 | 1716.46 | 907.25 |
| Calculation RT Day 4 Block 8 | 1736.82 | 671.49 | 1700.63 | 662.69 | 1714.79 | 882.92 |
| Calculation RT Day 5 Block 1 | 2039.61 | 1022.35 | 1913.01 | 891.64 | 1844.42 | 727.91 |
| Calculation RT Day 5 Block 2 | 1868.56 | 844.75 | 1763.75 | 741.07 | 1870.83 | 993.41 |
| Calculation RT Day 5 Block 3 | 1768.47 | 755.72 | 1751.51 | 614.78 | 1803.08 | 846.90 |
| Calculation RT Day 5 Block 4 | 1842.66 | 868.69 | 1846.78 | 783.46 | 1717.31 | 687.59 |
| Calculation RT Day 5 Block 5 | 1851.62 | 901.66 | 1731.57 | 651.50 | 1728.77 | 721.23 |
| Calculation RT Day 5 Block 6 | 1852.34 | 934.22 | 1681.16 | 667.63 | 1748.83 | 783.71 |
| Calculation RT Day 5 Block 7 | 1857.98 | 986.17 | 1661.54 | 683.29 | 1700.89 | 763.51 |
| Drill Accuracy Day 1 Block 1 | 0.95 | 0.06 | 0.95 | 0.05 | 0.92 | 0.08 |
| Drill Accuracy Day 1 Block 2 | 0.97 | 0.04 | 0.97 | 0.04 | 0.93 | 0.07 |
| Drill Accuracy Day 1 Block 3 | 0.96 | 0.04 | 0.96 | 0.05 | 0.95 | 0.06 |
| Drill Accuracy Day 1 Block 4 | 0.95 | 0.05 | 0.95 | 0.05 | 0.95 | 0.06 |
| Drill Accuracy Day 1 Block 5 | 0.96 | 0.05 | 0.96 | 0.06 | 0.94 | 0.09 |
| Drill Accuracy Day 2 Block 1 | 0.96 | 0.06 | 0.96 | 0.03 | 0.94 | 0.06 |
| Drill Accuracy Day 2 Block 2 | 0.97 | 0.04 | 0.95 | 0.06 | 0.94 | 0.08 |
| Drill Accuracy Day 2 Block 3 | 0.99 | 0.03 | 0.96 | 0.05 | 0.95 | 0.06 |
| Drill Accuracy Day 2 Block 4 | 0.96 | 0.04 | 0.94 | 0.06 | 0.98 | 0.02 |
| Drill Accuracy Day 2 Block 5 | 0.98 | 0.04 | 0.94 | 0.07 | 0.96 | 0.06 |
| Drill Accuracy Day 2 Block 6 | 0.98 | 0.03 | 0.98 | 0.04 | 0.97 | 0.05 |
| Drill Accuracy Day 3 Block 1 | 0.97 | 0.06 | 0.95 | 0.10 | 0.94 | 0.13 |
| Drill Accuracy Day 3 Block 2 | 0.96 | 0.03 | 0.94 | 0.07 | 0.95 | 0.07 |
| Drill Accuracy Day 3 Block 3 | 0.96 | 0.05 | 0.98 | 0.05 | 0.96 | 0.05 |
| Drill Accuracy Day 3 Block 4 | 0.96 | 0.05 | 0.97 | 0.04 | 0.97 | 0.04 |
| Drill Accuracy Day 3 Block 5 | 0.96 | 0.06 | 0.96 | 0.04 | 0.96 | 0.06 |
| Drill Accuracy Day 3 Block 6 | 0.97 | 0.05 | 0.97 | 0.05 | 0.96 | 0.05 |
| Drill Accuracy Day 3 Block 7 | 0.98 | 0.04 | 0.97 | 0.04 | 0.96 | 0.05 |
| Drill Accuracy Day 4 Block 1 | 0.94 | 0.07 | 0.94 | 0.09 | 0.95 | 0.09 |
| Drill Accuracy Day 4 Block 2 | 0.96 | 0.05 | 0.95 | 0.07 | 0.94 | 0.12 |
| Drill Accuracy Day 4 Block 3 | 0.95 | 0.07 | 0.97 | 0.05 | 0.98 | 0.04 |
| Drill Accuracy Day 4 Block 4 | 0.96 | 0.05 | 0.97 | 0.04 | 0.95 | 0.07 |
| Drill Accuracy Day 4 Block 5 | 0.95 | 0.07 | 0.97 | 0.06 | 0.96 | 0.04 |
| Drill Accuracy Day 4 Block 6 | 0.96 | 0.04 | 0.96 | 0.05 | 0.96 | 0.04 |
| Drill Accuracy Day 4 Block 7 | 0.95 | 0.07 | 0.96 | 0.05 | 0.94 | 0.05 |
| Drill Accuracy Day 4 Block 8 | 0.94 | 0.07 | 0.94 | 0.06 | 0.95 | 0.06 |
| Drill Accuracy Day 5 Block 1 | 0.86 | 0.16 | 0.90 | 0.08 | 0.87 | 0.18 |
| Drill Accuracy Day 5 Block 2 | 0.93 | 0.08 | 0.91 | 0.09 | 0.86 | 0.17 |
| Drill Accuracy Day 5 Block 3 | 0.93 | 0.07 | 0.94 | 0.07 | 0.90 | 0.11 |
| Drill Accuracy Day 5 Block 4 | 0.94 | 0.06 | 0.93 | 0.08 | 0.88 | 0.14 |
| Drill Accuracy Day 5 Block 5 | 0.92 | 0.07 | 0.94 | 0.08 | 0.91 | 0.10 |
| Drill Accuracy Day 5 Block 6 | 0.91 | 0.08 | 0.94 | 0.07 | 0.89 | 0.10 |
| Drill Accuracy Day 5 Block 7 | 0.93 | 0.09 | 0.92 | 0.06 | 0.90 | 0.12 |
| Calculation Accuracy Day 1 Block 1 | 0.92 | 0.08 | 0.91 | 0.07 | 0.91 | 0.09 |
| Calculation Accuracy Day 1 Block 2 | 0.94 | 0.05 | 0.94 | 0.06 | 0.95 | 0.06 |
| Calculation Accuracy Day 1 Block 3 | 0.92 | 0.08 | 0.93 | 0.06 | 0.92 | 0.06 |
| Calculation Accuracy Day 1 Block 4 | 0.94 | 0.05 | 0.94 | 0.08 | 0.93 | 0.09 |
| Calculation Accuracy Day 1 Block 5 | 0.93 | 0.06 | 0.92 | 0.08 | 0.91 | 0.08 |
| Calculation Accuracy Day 2 Block 1 | 0.92 | 0.08 | 0.95 | 0.06 | 0.89 | 0.08 |
| Calculation Accuracy Day 2 Block 2 | 0.90 | 0.06 | 0.93 | 0.10 | 0.91 | 0.08 |
| Calculation Accuracy Day 2 Block 3 | 0.95 | 0.08 | 0.94 | 0.06 | 0.93 | 0.06 |
| Calculation Accuracy Day 2 Block 4 | 0.93 | 0.07 | 0.92 | 0.08 | 0.90 | 0.12 |
| Calculation Accuracy Day 2 Block 5 | 0.93 | 0.06 | 0.92 | 0.13 | 0.90 | 0.08 |
| Calculation Accuracy Day 2 Block 6 | 0.93 | 0.07 | 0.93 | 0.07 | 0.90 | 0.09 |
| Calculation Accuracy Day 3 Block 1 | 0.89 | 0.08 | 0.93 | 0.06 | 0.91 | 0.08 |
| Calculation Accuracy Day 3 Block 2 | 0.90 | 0.17 | 0.94 | 0.06 | 0.92 | 0.07 |
| Calculation Accuracy Day 3 Block 3 | 0.95 | 0.05 | 0.93 | 0.07 | 0.92 | 0.08 |
| Calculation Accuracy Day 3 Block 4 | 0.92 | 0.05 | 0.93 | 0.08 | 0.92 | 0.10 |
| Calculation Accuracy Day 3 Block 5 | 0.93 | 0.07 | 0.96 | 0.06 | 0.95 | 0.06 |
| Calculation Accuracy Day 3 Block 6 | 0.95 | 0.05 | 0.94 | 0.07 | 0.94 | 0.07 |
| Calculation Accuracy Day 3 Block 7 | 0.92 | 0.09 | 0.96 | 0.06 | 0.91 | 0.07 |
| Calculation Accuracy Day 4 Block 1 | 0.92 | 0.07 | 0.94 | 0.08 | 0.92 | 0.06 |
| Calculation Accuracy Day 4 Block 2 | 0.92 | 0.08 | 0.92 | 0.08 | 0.91 | 0.07 |
| Calculation Accuracy Day 4 Block 3 | 0.94 | 0.05 | 0.92 | 0.08 | 0.92 | 0.06 |
| Calculation Accuracy Day 4 Block 4 | 0.93 | 0.07 | 0.91 | 0.11 | 0.89 | 0.14 |
| Calculation Accuracy Day 4 Block 5 | 0.94 | 0.06 | 0.95 | 0.06 | 0.91 | 0.08 |
| Calculation Accuracy Day 4 Block 6 | 0.93 | 0.07 | 0.94 | 0.06 | 0.93 | 0.06 |
| Calculation Accuracy Day 4 Block 7 | 0.94 | 0.06 | 0.95 | 0.07 | 0.91 | 0.08 |
| Calculation Accuracy Day 4 Block 8 | 0.93 | 0.09 | 0.94 | 0.07 | 0.92 | 0.09 |
| Calculation Accuracy Day 5 Block 1 | 0.94 | 0.05 | 0.95 | 0.06 | 0.87 | 0.16 |
| Calculation Accuracy Day 5 Block 2 | 0.91 | 0.12 | 0.93 | 0.06 | 0.90 | 0.09 |
| Calculation Accuracy Day 5 Block 3 | 0.95 | 0.07 | 0.96 | 0.06 | 0.91 | 0.11 |
| Calculation Accuracy Day 5 Block 4 | 0.94 | 0.07 | 0.96 | 0.05 | 0.93 | 0.07 |
| Calculation Accuracy Day 5 Block 5 | 0.94 | 0.06 | 0.96 | 0.05 | 0.93 | 0.08 |
| Calculation Accuracy Day 5 Block 6 | 0.92 | 0.07 | 0.95 | 0.05 | 0.92 | 0.07 |
| Calculation Accuracy Day 5 Block 7 | 0.92 | 0.08 | 0.96 | 0.06 | 0.94 | 0.05 |
