# Supplementary material for: Functional connectivity and GABAergic signaling modulate the enhancement effect of neurostimulation on mathematical learning
Source: PLoS Biol. 2025 Jul 1;23(7):e3003200. doi: 10.1371/journal.pbio.3003200 (PMC12212564; doi:10.1371/journal.pbio.3003200)
Supplement: S13 Table — (DOCX) [file pbio.3003200.s019.docx]

**S13 Table**. Minimum Reporting Standards for MRS in MRS checklist.

1. Hardware

| Field strength [T] | 3 T |
| --- | --- |
| Manufacturer | Siemens |
| Model | Verio |
| RF coils: nuclei (transmit/receive), number of channels, type, body part | 32-channel receive head coil |
| Additional hardware | N/A |

1. Acquisition

| Pulse sequence | SPECIAL (spin-echo full-intensity acquired localized) |
| --- | --- |
| Voxel of interest (VOI) location | Manually localized on axial and coronal slices and placed over the left PPC and the left dlPFC and primary visual cortex (V1) |
| Nominal voxel size | 2x2x2cm^3^ for all voxels |
| TR/TE [ms] | 4000/8.5ms |
| Total number of excitations or acquisitions per spectrum | Water suppressed: 128 averages  Non-water-suppressed: 8 averages |
| In time series for kinetic studies   1. Number of averaged spectra (NA) per time point 2. Averaging method 3. Total number of spectra (acquired/in time series) | N/A |
| Additional sequence parameters (spectral width [Hz], number of spectral points, frequency offset), 2D FOV, matrix size, acceleration factors, sampling method | Spectral width: 4000Hz  Number of spectral points: 4096  Frequency offset: -2.3 ppm |
| Water suppression method | VAPOR (variable power radio frequency pulses with optimized relaxation delays) |
| Shimming method | GRE-shim method, i.e., the Siemens "Brain" shim mode |
| Triggering or motion correction method | N/A |

1. Data analysis methods and outputs

| Analysis software | FID-A for data processing and LCModel for metabolite quantification |
| --- | --- |
| Processing steps deviating from quoted reference or product | -LCModel default MM basis spectra were replaced with in-house MM basis spectra.  -Other LCModel parameters of interest:   - PPMST=0.2 - PPMEND=4.2 - DKNTMN = 0.25 |
| Output measure | Absolute concentrations were corrected for GM, WM and CSF volume fraction, with assumed T1 and T2 of water within each tissue type, and assumed T1 and T2 of metabolites within the tissue (GM+WM). The formula for absolute quantification is provided in the manuscript. Uncertainties reported as %CRLB. |
| Quantification references and assumptions, fitting model assumptions | For LCModel, the unsuppressed water signal was used as an internal reference for metabolite quantification. The unsuppressed water scan was also used for eddy current correction in LCModel. |

1. Data quality

| Reported variables (SNR, linewidth (with reference peaks)) | For spectra plots and LCmodel fit estimates see **S5 Fig**. |
| --- | --- |
| Data exclusion criteria | - LCModel-reported linewidth > 8 Hz. - SNR below 3 x study SD per time-point, per region - Glutamate or GABA concentration not quantified by LCModel. - Metabolite concentration beyond 3 SD per metabolite (GABA or Glutamate) per time point, per region. |
| Quality measures of postprocessing model fitting (e.g. CRLB, goodness of fit, SD of residual) | SNR, LW |
| Sample spectrum | For spectra plots and LCmodel fit estimates see **S5 Fig**. |
