# Supplementary material for: Functional connectivity and GABAergic signaling modulate the enhancement effect of neurostimulation on mathematical learning
Source: PLoS Biol. 2025 Jul 1;23(7):e3003200. doi: 10.1371/journal.pbio.3003200 (PMC12212564; doi:10.1371/journal.pbio.3003200)
Supplement: Attachment — Submitted filename: Response to Reviewers.docx [file pbio.3003200.s026.docx]

Dear Dr Schnell,

We would like to thank you and the reviewers for your positive and constructive responses to our manuscript titled “Unveiling Causal Mechanisms of Academic Learning Through Neurostimulation and Multimodal Imaging” (**PBIOLOGY-D-24-02991R1**). We have carefully revised the manuscript and addressed all the issues that were raised during the review process (italicized).

We hope that you and the reviewers will find our response satisfactory and that the revised manuscript is much improved.

Sincerely,

Dr George Zacharopoulos *et al.*

**Reviewer #1.** We are grateful to the reviewer for her/his overall positive evaluation of our work. The main comments raised by the reviewer were:

**R1.C1.** *Heuristic Nature of the Manuscript: The entire manuscript is written in a highly heuristic manner. The interpretations of the results are not grounded in clear hypotheses or predictions derived from established models. Instead, interpretations appear speculative, lacking further experiments or theoretical frameworks to support them. This makes the manuscript overly speculative, reducing its scientific rigor. Lack of Hypotheses and Counter-Hypotheses: While hypotheses and predictions are mentioned, the manuscript does not explain why these specific hypotheses or predictions were made. Furthermore, no counter-hypotheses are discussed, which would strengthen the interpretation of the results and demonstrate a more balanced approach. Recommendations for Improvement: Elaborate on Hypotheses and Predictions: Clearly state the hypotheses and predictions, ensuring they are derived from prior experimental results, theoretical models, or logical reasoning. Including counter-hypotheses would also provide a more comprehensive framework for interpreting the results. Speculative Conclusions: It feels as though the multimodal approach yielded interesting results, but these findings were then used as a basis for speculation rather than being tied to robust experimental evidence or theoretical models.*

**R1.R1.** We thank the reviewer for their comment. As suggested, we have now significantly expanded the Introduction by clearly explaining the rationale of the hypotheses using prior experimental results and theoretical models.

The Introduction now states: “Neurocognitive models have suggested that arithmetic information is processed in a multimodal and distributed manner in the human brain. Accounts based on Bayesian meta-analytic models identified the canonical circuit of arithmetic problem solving (1) which consists of brain regions that are mostly localized to the dorsal aspects of the posterior parietal cortex (PPC), ventral temporal-occipital (VTOC), and the prefrontal cortex (PFC), including the premotor cortex, the inferior and middle frontal gyri (2).

Previous accounts for how arithmetic information is mastered indicated that the development of numerical and arithmetic abilities is enabled by a significant increase in working memory (3). Indeed, the role of working memory and executive functions is a crucial feature in several theoretical models of cognitive learning in general and arithmetic learning in particular (A Triarchic Theory of Learning, (4)). These models predict that mathematical procedures (calculation), which rely heavily on executive functions including working memory, attention processes, inhibition, and shifting (5) are typically associated with the dlPFC (6). This view is supported by previous work on the acquisition of arithmetic expertise in normally developing children and adults revealed that a positive shift in arithmetic competence corresponds to a shift from effortful processing that engages the prefrontal regions, such as the dlPFC to skilled and fast retrieval that involves parietal regions such as the PPC (7). This is because repeating the arithmetic problems strengthens a direct retrieval of the correct solution from memory (e.g., 23 × 8 = 184). Concurrently, the demand on quantity-based processing and complex processes, such as working memory (e.g., 23 × 8 = (20 × 8) + (3 × 8) = 160 + 24 = 184), monitoring and attention, sustained by frontal areas, is diminished (7). Accordingly, we hypothesize that tRNS over the dlPFC, while participants learn to apply a new algorithm, will improve calculation learning. Based on a previous tRNS study using the same paradigm as in this work we predicted that the effect will be found for reaction times, rather than accuracy (8). In contrast, we did not expect to find a reliable tRNS effect for drill learning, which does not require the application of mathematical procedures. However, tRNS over the PPC may improve drill learning, but not calculation learning. While in a previous study no effect was found when tRNS was applied over the PPC during calculation and drill learning (8), a vocabulary learning task, which involves some component of drill learning (9) has shown an improvement following the same parietal montage and tRNS parameters as in our study (8).

Apart from the aforementioned dissociated roles of the frontal and the parietal regions, these regions, which are co-activated during arithmetic tasks (2), form the frontoparietal network that support the manipulation of numerical information which is crucial calculation learning. Apart from frontoparietal networks, the frontohippocampal networks are involved in creating associative memories and connecting new information with existing knowledge, which contributes to the development of long-term memories that enable generalization beyond specific problem characteristics (10). Taking these results, together with previous work showing that cortico-hippocampal connectivity is involved in mathematical learning (11), we hypothesize that frontoparietal connectivity will be associated mainly with calculation learning, while frontohippocampal connectivity will be associated with both drill and calculation learning performance.

Beyond the brain regions and networks involved in mathematical learning, previous studies, have highlighted the crucial role of excitatory and inhibitory neural circuits in shaping neural excitation/inhibition (E/I) balance. E/I balance governs the onset and closure of sensitive periods in brain development and skill acquisition (12-14). For example, in humans, research on visual procedural learning has utilized glutamate and GABA measurements to quantify E/I balance, associating high E/I with plasticity (the capacity for change in learning mechanisms) and lower E/I with stability (the resistance to change in these mechanisms) (15). Effective learning requires an initial phase of plasticity, followed by a phase of stability to consolidate knowledge and prevent interference from subsequent learning experiences (15). However, the role of plasticity and stability in more complex learning domains, such as mathematics, remains underexplored (16). In this study, we employed tRNS due to its potential to change the levels of excitation and inhibition (16-19). Similar to previous neuroimaging studies we noninvasively quantified excitation and inhibition using glutamate and GABA, respectively (20-22). tRNS, due to its potential excitatory effect, has been suggested to enhance plasticity with a continuous effect beyond the stimulation session, as indicated by lasting effects beyond the stimulation period (e.g., weeks to months; (8, 9, 17, 23-27). Our approach is further motivated by the association between individual variability in glutamate and GABA levels and mathematical attainment (28). Therefore, we hypothesize that changes in glutamate and GABA levels will predict calculation and drill learning.” (line=13, paragraph=3, page=4)

Moreover, we have now significantly expanded the Discussion by grounding the interpretation of the results in clear hypotheses/predictions derived from established models of arithmetic cognition. The Discussion now states: “This study provides novel insights into the neural mechanisms underlying mathematical learning, specifically in terms of the role of dlPFC in calculation but not drill learning in adults. Namely, in contrast to previous fMRI studies that highlighted the role of dlPFC also in drill learning, our findings suggest that dlPFC may instead be a correlate and may indicate redundancy in brain functions (29) with regards to drill learning in the adult brain. While we included a mathematical control task, future studies should include a non-mathematical learning control task that also heavily depends on executive functions to help distinguish between specific executive function components. This will allow the examination of whether the observed neurostimulation effect is specific to mathematical learning or due to a domain-general modulation of executive functions. Nevertheless, our findings are theoretically consistent with current meta-analyses and models of arithmetic processing (6, 10, 30-34). Our two-time point (i.e., before vs after tRNS) additional analyses did not provide evidence that tRNS modulated working memory/executive functions (**Supporting Information 16**). Recent tRNS experiments that used a similar prefrontal montage as in this study, improved sustained attention performance or symptoms that are associated with sustained attention impairments (17, 24, 35). Therefore, we hypothesize that our results are due to sustained attention enhancement that has been linked with improved academic learning (36). In this case, the lack of effect on drill learning may be explained by task difficulty. This is in line with previous tRNS studies that have shown that in line with the stochastic resonance framework, task difficulty moderates the effect of tRNS (37, 38). Namely, the presence of a dlPFC-tRNS effect for calculation learning might not necessarily be due to the fact the dlPFC is less involved in drill learning, but because drill is an easy task and less demanding compared to calculation. Highly task engagement is thought to boost the effectiveness of non-invasive brain stimulation (39). Based on the stochastic resonance framework (37) it is expected that the more difficult the task the more the benefit from tRNS. Our findings, which are in line with previous work in the field of cognitive learning (16, 38), match these predictions.” (line=22, paragraph=2, page=22)

**R1.C2.** *Recommendations for Improvement: Conduct Additional Experiments: Perform further experiments to validate the speculations derived from the current findings. This would lend greater credibility to the interpretations and strengthen the manuscript's overall contribution.*

**R1.R2.** As requested, we have now conducted further analyses to test the potential explanations derived from the current findings. Specifically, we examined the effect of tRNS on modulating verbal and visuospatial short-term memory and working memory and the executive and orienting scores from the attention network task. We now present these results in **Supporting Information 16** which we paste below.

**Supporting Information 16.** Independent sample t-test comparing the sham condition vs dlPFC-tRNS (**Supporting Information 16A**) or vs PPC-tRNS (**Supporting Information 16B**) for the following scores: [Attention scores derived by the Attention Network Task (40) where Executive: ANT Executive network, Orienting: ANT Orienting network; Short term memory assessed by the Digit span task (41) and the Corsi Block tapping task (42) tests that included both forward and backward conditions]. A “Δ” prefix denotes a post-tRNS minus pre-tRNS difference score in each cognitive measure.

|  | **T** | **DF** | **P** | **Mean** | **SE** | **CI_L** | **CI_U** |
| --- | --- | --- | --- | --- | --- | --- | --- |
| **Supporting Information 16A** | | | | | | | |
| Δ Executive | 0.1 | 45 | 0.897 | 0.9 | 6.8 | -12.7 | 14.5 |
| Δ Orienting | -0.9 | 46 | 0.360 | -6.8 | 7.4 | -21.7 | 8.0 |
| Δ digitSpanFwd | -0.3 | 42 | 0.789 | -0.2 | 0.6 | -1.3 | 1.0 |
| Δ digitSpanBwd | 1.6 | 40 | 0.127 | 1.0 | 0.6 | -0.3 | 2.2 |
| Δ CorsiBwd | 0.5 | 39 | 0.593 | 0.3 | 0.5 | -0.8 | 1.4 |
| Δ CorsiFwd | -0.6 | 39 | 0.531 | -0.4 | 0.7 | -1.7 | 0.9 |
|  |  |  |  |  |  |  |  |
| **Supporting Information 16B** | | | | | | | |
| Δ Executive | 0.0 | 45 | 0.971 | -0.2 | 6.4 | -13.2 | 12.7 |
| Δ Orienting | 0.7 | 46 | 0.476 | 5.1 | 7.1 | -9.1 | 19.3 |
| Δ digitSpanFwd | -1.5 | 41 | 0.135 | -1.0 | 0.6 | -2.2 | 0.3 |
| Δ digitSpanBwd | 0.6 | 39 | 0.576 | 0.4 | 0.6 | -0.9 | 1.7 |
| Δ CorsiBwd | 0.3 | 37 | 0.783 | 0.1 | 0.4 | -0.8 | 1.0 |
| Δ CorsiFwd | 0.3 | 38 | 0.767 | 0.2 | 0.6 | -1.1 | 1.4 |

**Reviewer #2.** We are grateful to the reviewer for her/his overall positive evaluation of our work. The main comments raised by the reviewer were:

**R2.C1.** *I note the often change in MRS measured metabolite levels are described as "neurotransmitter" levels. This is not accurate. Please adjust this language throughout. Please define what exactly being is measured using MRS (cellular/synaptic/extracellular) to give the general reader a clearer understanding. Also please expand upon why MRS measured glutamate and GABA can be taken as a proxy for E/I - at what levels? I'd suggest including with appropriate references the evidence that the MRS substrate quantified may be a reasonable reflection of neurotransmitter levels (there are preclinical studies to this effect) and this can subsequently* *support discussion of results in terms of a neurosignalling response.*

**R2.R1.** As suggested, we have now adjusted the language throughout the manuscript by changing the term “neurotransmitter” to “neurochemical”. Moreover, we now define what is being measured using MRS and include appropriate references from preclinical studies that evidence that the MRS substrate quantified is a reasonable reflection of neurotransmitter levels. The text now reads: “Please note that MRS alone cannot distinguish between the different pools of a given neurochemical, namely, MRS will detect the presence of a neurochemical regardless of the precise location which can be cellular, synaptic or extracellular. However, prior animal and human preclinical studies indicated that MRS-quantified levels of GABA and glutamate are a reasonable reflection of neurotransmitter levels and thus can be taken as a proxy for Excitation/Inhibition balance. Specifically, region-specific changes in GABA and glutamate assessed with MRS may reflect a shift in the balance of excitatory/inhibitory transmission in the context of preclinical studies including ageing, inflammation, and neurodegeneration neurotransmission (43).” (line=10, paragraph=2, page=35).

**R2.C2.** *Still on MRS - could the manuscript please be updated to confirm with MRS reporting guidelines - especially as they have a co-author of this guidance.*

**R2.R2.** We have updated the manuscript to conform with MRS reporting guidelines (44) and present this in **Supporting Information 18**, which we now paste below.

**Supporting Information 18**: Minimum Reporting Standards for MRS in MRS checklist.

1. Hardware

| Field strength [T] | 3 T |
| --- | --- |
| Manufacturer | Siemens |
| Model | Verio |
| RF coils: nuclei (transmit/receive), number of channels, type, body part | 32-channel receive head coil |
| Additional hardware | N/A |

2. Acquisition

| Pulse sequence | SPECIAL (spin-echo full-intensity acquired localized) |
| --- | --- |
| Volume of interest (VOI) location | Manually localized on axial and coronal slices and placed over the left PPC and the left dlPFC and primary visual cortex (V1) |
| Nominal voxel size | 2x2x2cm^3^ for all voxels |
| TR/TE [ms] | 4000/8.5ms |
| Total number of excitations or acquisitions per spectrum | Water suppressed: 128 averages  Non-water-suppressed: 8 averages |
| In time series for kinetic studies   i. Number of averaged spectra (NA) per time point ii. Averaging method iii. Total number of spectra (acquired/in time series) | N/A |
| Additional sequence parameters (spectral width [Hz], number of spectral points, frequency offset), 2D FOV, matrix size, acceleration factors, sampling method | Spectral width: 4000Hz  Number of spectral points: 4096  Frequency offset: -2.3 ppm |
| Water suppression method | VAPOR (variable power radio frequency pulses with optimized relaxation delays) |
| Shimming method | GRE-shim method, i.e., the Siemens "Brain" shim mode |
| Triggering or motion correction method | N/A |

3. Data analysis methods and outputs

| Analysis software | FID-A for data processing and LCModel for metabolite quantification |
| --- | --- |
| Processing steps deviating from quoted reference or product | -LCModel default MM basis spectra were replaced with in-house MM basis spectra.  -Other LCModel parameters of interest:   - PPMST=0.2 - PPMEND=4.2 - DKNTMN = 0.25 |
| Output measure | Absolute concentrations were corrected for GM, WM and CSF volume fraction, with assumed T1 and T2 of water within each tissue type, and assumed T1 and T2 of metabolites within the tissue (GM+WM). The formula for absolute quantification is provided in the manuscript. Uncertainties reported as %CRLB. |
| Quantification references and assumptions, fitting model assumptions | For LCModel, the unsuppressed water signal was used as an internal reference for metabolite quantification. The unsuppressed water scan was also used for eddy current correction in LCModel. |

4. Data quality

| Reported variables (SNR, linewidth (with reference peaks)) | For spectra plots and LCmodel fit estimates see **Supporting Information 13**. |
| --- | --- |
| Data exclusion criteria | - LCModel-reported linewidth > 8 Hz. - SNR below 3 x study SD per time-point, per region - Glutamate or GABA concentration not quantified by LCModel. - Metabolite concentration beyond 3 SD per metabolite (GABA or Glutamate) per time point, per region. |
| Quality measures of postprocessing model fitting (eg CRLB, goodness of fit, SD of residual) | SNR, LW |
| Sample spectrum | For spectra plots and LCmodel fit estimates see **Supporting Information 13**. |

**Reviewer #3.** We are grateful to the reviewer for her/his overall positive evaluation of the contribution of our findings in the literature. The main comments raised by the reviewer were:

**R3.C1.** *The authors find significant effects exclusively for mathematical procedures (e.g., the relationship between stronger connectivity and learning success, or between frontal tRNS and learning success) but not with memory-based drill training. Mathematical procedures in arithmetic heavily rely on EF like working memory, attention processes, inhibition, or shifting (see Cragg & Gilmore, 2014 for a review), typically assumed to be associated with the DLPFC. In line with this, the modulation observed in this study occurred only with tRNS over the DLPFC. However, the study lacks a non-mathematical control task that also heavily depends on EF, let alone control tasks that might help distinguish between specific EF components. As a result, it cannot be concluded that the observed effect is specific to mathematical learning.
Despite this, the authors draw precisely this conclusion (p. 19): "This study provides novel insights into the neural mechanisms underlying mathematical learning." An alternative interpretation would be that the findings represent a domain-general modulation of EF, which does not need to be inherently specific to mathematical learning. This is not necessarily a limitation; in fact, the results might suggest implications for EF trainings, which in turn could indirectly enhance mathematical procedures. Nevertheless, the results should be interpreted and discussed more cautiously.*

*That said, the authors could emphasize more that their findings are nevertheless theoretically consistent with current meta-analyses and models of numerical processing. According to these models, mathematical procedures and calculation impose high demands on EF, reflected in strong frontal activation within the math-responsive network. Meanwhile, encoding arithmetic facts is supposed to be stronger associated with medial temporal areas, as well as inferior parietal and temporal cortices (e.g., Amalric & Dehaene, 2018; Arsalidou & Taylor, 2011; Arsalidou et al., 2018; Hawes et al., 2019; Klein & Knops, 2023; Menon, 2016; Siemann & Petermann, 2018).*

*This opens up an alternative explanation for the absence of effects during drill training. Instead of interpreting this as "It might indicate that the effect of tRNS on mathematical drill learning is less robust," it could simply be that other brain regions critical for drill training—such as the hippocampus—were not targeted by tRNS in this study (which, indeed, cannot be stimulated directly).*

**R3.R1.** We thank the reviewer for their comment. We would like to clarify that at no place did we conclude that the observed effect is specific to mathematical learning. This was not the aim of our study. Our conclusion that  *"This study provides novel insights into the neural mechanisms underlying mathematical learning."* stands by itself without the need to conclude about the specificity of the effect. However, we do conclude that the effect is not general. Namely, it cannot be attributed to non-specific factors such as motivation or placebo.

Nevertheless, to avoid similar confusion by other readers we have now clarified these issues. The Discussion now states: “This study provides novel insights into the neural mechanisms underlying mathematical learning, specifically in terms of the role of dlPFC in calculation but not drill learning in adults. Namely, in contrast to previous fMRI studies that highlighted the role of dlPFC also in drill learning, our findings suggest that dlPFC may instead be a correlate and may indicate redundancy in brain functions (29) with regards to drill learning in the adult brain. While we included a mathematical control task, future studies should include a non-mathematical learning control task that also heavily depends on executive functions to help distinguish between specific executive function components. This will allow the examination of whether the observed neurostimulation effect is specific to mathematical learning or due to a domain-general modulation of executive functions. Nevertheless, our findings are theoretically consistent with current meta-analyses and models of arithmetic processing (6, 10, 30-34). Our two-time point (i.e., before vs after tRNS) additional analyses did not provide evidence that tRNS modulated working memory/executive functions (**Supporting Information 16**). Recent tRNS experiments that used a similar prefrontal montage as in this study, improved sustained attention performance or symptoms that are associated with sustained attention impairments (17, 24, 35). Therefore, we hypothesize that our results are due to sustained attention enhancement that has been linked with improved academic learning (36). In this case, the lack of effect on drill learning may be explained by task difficulty. This is in line with previous tRNS studies that have shown that in line with the stochastic resonance framework, task difficulty moderates the effect of tRNS (37, 38). Namely, the presence of a dlPFC-tRNS effect for calculation learning might not necessarily be due to the fact the dlPFC is less involved in drill learning, but because drill is an easy task and less demanding compared to calculation. Highly task engagement is thought to boost the effectiveness of non-invasive brain stimulation (39). Based on the stochastic resonance framework (37) it is expected that the more difficult the task the more the benefit from tRNS. Our findings, which are in line with previous work in the field of cognitive learning (16, 38), match these predictions.” (line=22, paragraph=2, page=22)

We agree with the reviewer that there are other alternative explanations for the absence of tRNS effects on drill learning. This may be due to the fact that the current study did not target the hippocampus, which may be critical for drill training as suggested by several existing models of mathematical cognition (10, 33), but see (45). This is because subcortical regions cannot be directly targeted by tRNS. Recent advances in non-invasive brain stimulation techniques allow us to examine the role of the hippocampus in drill learning using emerging technologies (e.g., transcranial ultrasound or temporal interference stimulation) to precisely target subcortical structures such as the hippocampus. Such emerging techniques are particularly relevant for advancing our understanding of key aspects of mathematical cognition that are subserved by subcortical structures and by the interactions with the cortex (46, 47).

**R3.C2.** *There are other conclusions that are not justified in my opinion. The authors conclude (p. 15): "Our results, in line with previous results (6-8), support the view that such differences are primarily of biological, rather than environmental, origin. Importantly, our results highlight an approach that can reverse such disadvantage using an excitatory form of neurostimulation to target the neural substrates involved in calculation learning to abolish the Matthew effect." These claims are problematic for several reasons: 1) The study does not provide evidence to definitively determine whether differences in learning mathematical procedures are of biological rather than environmental origin as this was not tested in the study. To draw such a causal conclusion, environmental factors, such as socioeconomic status (SES), would need not only to be controlled for but also systematically manipulated (e.g., through the inclusion of an additional group). 2) The claim that the study
demonstrates the ability of tRNS to "reverse" disadvantages associated with the Matthew effect is also unfounded. On the one hand, this presupposes a long-term effect, which was not tested here and therefore cannot be assumed. The measurements were conducted immediately after the five-day training, and potential long-term effects of electrical stimulation are, at best, highly debated. On the other hand, this presupposes that the improvement effects are within a considerable range that has real practical relevance (ideally not only within the ms range of reaction times, but also relevant accuracy improvements etc.) instead of rather academic relevance (for the elicitation of model-theoretical questions, however, this study is definitely novel and interesting). However, I could not find accuracy data in the supplementary materials (SM), nor data plots on training curves (e.g., fewer repetitions during training could have been an interesting indicator). Even the reaction time
data analysis is hidden in Figures S6A and S6B, and these effects do not appear particularly large. Therefore, I would tone down the claims regarding practical relevance. Together with the first major point, the correction of this discussion and conclusions requires, in my opinion, reframing of the manuscript.*

**R3.R2.** We would like to thank the reviewer for these comments. As for the Matthew effect, previous studies did not argue that the effect should be assessed over a long period of time. While we tested the effect of learning immediately, the observed effects are in line with the Matthew effect. For example, the Matthew effect was also demonstrated in several working memory training interventions, whereby those with initial higher ability benefited more from training on this ability as well as on new skills (48-51). In these studies, the post-measurements were temporally identical to the end of training, like in the present study, or very temporal proximity (i.e., one week). In any case, we have now addressed the key points raised. First we have amended this initial term “ever-increasing inequality” to “even greater increase in inequality” to reflect our conclusions more accurately. Accordingly, the text now states, “The Matthew effect (52) has been shown in different types of learning, including mathematical learning (53), language learning (54, 55), and memory-related processes such as vocabulary growth (55), whereby a baseline cognitive gap between high and low achievers leads to an even greater increase in inequality between these two groups.” (line=10, paragraph=1, page=17)

Second, the text now states: "Our results, in line with previous results (56-58), support the view that such differences may stem from biological origin. However, such differences may be independently influenced by socioeconomic status and polygenetic factors (59). By using a learning task and modulating the neural correlate involved in mathematical learning we were able to show that the Matthews effect can be explained and mitigated at the biological level. Our results do not undermine the environmental factors (e.g., socioeconomic status) that could also draw a causal effect but are less likely to play a role in this study due to the random allocation of participants, and using a learning task. Our results highlight the possibility of reversing such biological disadvantage using an excitatory form of neurostimulation to target the neural substrates involved in calculation learning. This is in line with a recent study that identified genes that correlate with mathematical abilities, as crucial for neuronal excitability (58). Future studies can build on our findings by assessing how long this effect lasts, and whether such an approach can have practical relevance outside the controlled lab environment. Answering these questions would have important implications for the fields of education and science of learning.” (line=14, paragraph=2, page=17)

In addition, we have conducted the analyses at the accuracy level that we now present in **Supporting Information 14**-**15**. As in our original study (8), there was no effect on accuracy. This can be explained by our paradigm that emphasises accuracy, therefore increasing the sensitivity to detect reaction times effect (60). We have pasted **Supporting Information 14**-**15** below.

**Supporting Information 14.** Additional statistical analyses examining the role of baseline frontoparietal connectivity in predicting accuracy. **Statistics:** Value= regression coefficient, SE=standard error, DF=degrees of freedom, T=T-value, P=P-value, CI_L=confidence interval lower bound, CI_U=confidence interval upper bound. Interactor predictors are denoted by the * symbol.

|  | **Value** | **SE** | **DF** | **T** | **P** | **CI_L** | **CI_U** |
| --- | --- | --- | --- | --- | --- | --- | --- |
| **Supporting Information 14A (Right Hemisphere)** | | | | | | | |
| (Intercept) | 0.95 | 0.03 | 210 | 34.81 | 0.0000 | 0.89 | 1.00 |
| Day | 0.00 | 0.01 | 210 | 0.43 | 0.6679 | -0.01 | 0.02 |
| TypeDrill | 0.04 | 0.03 | 210 | 1.43 | 0.1547 | -0.02 | 0.10 |
| Frontoparietal_R | -0.04 | 0.04 | 22 | -0.98 | 0.3378 | -0.12 | 0.04 |
| Day*TypeDrill | -0.02 | 0.01 | 210 | -2.05 | 0.0419 | -0.04 | 0.00 |
| Day*Frontoparietal_R | 0.00 | 0.01 | 210 | -0.19 | 0.8514 | -0.02 | 0.02 |
| TypeDrill*Frontoparietal_R | 0.03 | 0.04 | 210 | 0.74 | 0.4595 | -0.05 | 0.11 |
| Day*TypeDrill*Frontoparietal_R | 0.01 | 0.01 | 210 | 0.76 | 0.4477 | -0.02 | 0.03 |
| **Supporting Information 14B (Left Hemisphere)** | | | | | | | |
| (Intercept) | 0.90 | 0.03 | 210 | 25.63 | 0.0000 | 0.83 | 0.96 |
| Day | 0.00 | 0.01 | 210 | -0.09 | 0.9273 | -0.02 | 0.02 |
| TypeDrill | 0.11 | 0.04 | 210 | 2.72 | 0.0071 | 0.03 | 0.18 |
| Frontoparietal_L | 0.04 | 0.05 | 22 | 0.80 | 0.4337 | -0.07 | 0.15 |
| Day*TypeDrill | -0.02 | 0.01 | 210 | -1.92 | 0.0556 | -0.05 | 0.00 |
| Day*Frontoparietal_L | 0.00 | 0.01 | 210 | 0.30 | 0.7662 | -0.02 | 0.03 |
| TypeDrill*Frontoparietal_L | -0.07 | 0.06 | 210 | -1.16 | 0.2491 | -0.18 | 0.05 |
| Day*TypeDrill*Frontoparietal_L | 0.02 | 0.02 | 210 | 0.94 | 0.3462 | -0.02 | 0.05 |

**Supporting Information 15.** A table depicting the statistical results of the linear mixed-effects model predicting accuracy based on learning type (drill, calculation), tRNS condition (sham tRNS which is the reference group here, dlPFC-tRNS), dlPFC GABA concentration, day, and right frontoparietal connectivity. Statistics: Value=regression coefficient, SE=standard error, DF=degrees of freedom, T=T-value, P=P-value. Interactor predictors are denoted by the * symbol. Neurochemicals: dlPFC= dorsolateral prefrontal cortex, GABA= gamma-aminobutyric acid. A “Δ” prefix denotes a post-tRNS minus pre-tRNS difference score in that neurochemical or functional connectivity measure.

|  | **Value** | **SE** | **DF** | **T** | **P** | **CI_L** | **CI_U** |
| --- | --- | --- | --- | --- | --- | --- | --- |
| (Intercept) | 0.95 | 0.03 | 120 | 32.15 | 0.00 | 0.89 | 1.01 |
| Day | 0.00 | 0.01 | 120 | 0.27 | 0.79 | -0.01 | 0.02 |
| TypeDrill | 0.03 | 0.03 | 120 | 1.02 | 0.31 | -0.03 | 0.10 |
| Δ FC | -0.01 | 0.06 | 8 | -0.23 | 0.82 | -0.16 | 0.13 |
| Δ dlPFC GABA | 0.19 | 0.18 | 8 | 1.02 | 0.34 | -0.24 | 0.61 |
| DlPFC-tRNS | -0.02 | 0.03 | 8 | -0.53 | 0.61 | -0.09 | 0.06 |
| Day*TypeDrill | 0.00 | 0.01 | 120 | 0.05 | 0.96 | -0.02 | 0.02 |
| Day*Δ FC | 0.01 | 0.01 | 120 | 0.52 | 0.61 | -0.02 | 0.04 |
| TypeDrill*Δ FC | 0.06 | 0.07 | 120 | 0.86 | 0.39 | -0.08 | 0.20 |
| Day*Δ dlPFC GABA | -0.01 | 0.04 | 120 | -0.30 | 0.77 | -0.10 | 0.07 |
| TypeDrill*Δ dlPFC GABA | -0.21 | 0.20 | 120 | -1.05 | 0.29 | -0.61 | 0.19 |
| Δ FC*Δ dlPFC GABA | 0.13 | 0.51 | 8 | 0.25 | 0.81 | -1.05 | 1.30 |
| Day*DlPFC-tRNS | 0.00 | 0.01 | 120 | 0.53 | 0.60 | -0.01 | 0.02 |
| TypeDrill*DlPFC-tRNS | 0.03 | 0.04 | 120 | 0.79 | 0.43 | -0.04 | 0.10 |
| Δ FC*DlPFC-tRNS | -0.02 | 0.08 | 8 | -0.25 | 0.81 | -0.21 | 0.17 |
| Δ dlPFC GABA*DlPFC-tRNS | -0.17 | 0.19 | 8 | -0.88 | 0.40 | -0.62 | 0.28 |
| Day*TypeDrill*Δ FC | -0.03 | 0.02 | 120 | -1.20 | 0.23 | -0.07 | 0.02 |
| Day*TypeDrill*Δ dlPFC GABA | 0.06 | 0.06 | 120 | 0.95 | 0.34 | -0.06 | 0.18 |
| Day*Δ FC*Δ dlPFC GABA | -0.09 | 0.12 | 120 | -0.74 | 0.46 | -0.33 | 0.15 |
| TypeDrill*Δ FC*Δ dlPFC GABA | -0.05 | 0.56 | 120 | -0.09 | 0.93 | -1.17 | 1.06 |
| Day*TypeDrill*DlPFC-tRNS | -0.02 | 0.01 | 120 | -1.40 | 0.16 | -0.04 | 0.01 |
| Day*Δ FC*DlPFC-tRNS | -0.01 | 0.02 | 120 | -0.42 | 0.67 | -0.05 | 0.03 |
| TypeDrill*Δ FC*DlPFC-tRNS | -0.02 | 0.09 | 120 | -0.18 | 0.86 | -0.19 | 0.16 |
| Day*Δ dlPFC GABA*DlPFC-tRNS | 0.02 | 0.05 | 120 | 0.40 | 0.69 | -0.07 | 0.11 |
| TypeDrill*Δ dlPFC GABA*DlPFC-tRNS | 0.24 | 0.21 | 120 | 1.12 | 0.27 | -0.19 | 0.66 |
| Δ FC*Δ dlPFC GABA*DlPFC-tRNS | -0.36 | 0.52 | 8 | -0.68 | 0.52 | -1.56 | 0.85 |
| Day*TypeDrill*Δ FC*Δ dlPFC GABA | -0.02 | 0.17 | 120 | -0.09 | 0.93 | -0.35 | 0.32 |
| Day*TypeDrill*Δ FC*DlPFC-tRNS | 0.01 | 0.03 | 120 | 0.50 | 0.62 | -0.04 | 0.07 |
| Day*TypeDrill*Δ dlPFC GABA*DlPFC-tRNS | -0.08 | 0.06 | 120 | -1.18 | 0.24 | -0.20 | 0.05 |
| Day*Δ FC*Δ dlPFC GABA*DlPFC-tRNS | 0.11 | 0.12 | 120 | 0.86 | 0.39 | -0.14 | 0.35 |
| TypeDrill*Δ FC*Δ dlPFC GABA*DlPFC-tRNS | 0.28 | 0.58 | 120 | 0.48 | 0.63 | -0.87 | 1.42 |
| Day*TypeDrill*Δ FC*Δ dlPFC GABA*DlPFC-tRNS | -0.06 | 0.17 | 120 | -0.32 | 0.75 | -0.40 | 0.29 |

**R3.C3.** *Since „stronger baseline positive connectivity" showed better learning performance in this study, I am missing a discussion with previous data showing detrimental influence of stronger functional connectivity in participants with lower mathematical abilities (e.g., Rosenberg-Lee et al., 2015; Jolles et al., 2016; Abreu-Mendoza et al. 2022; Michels et al., 2018).*

**R3.R3.** As suggested, we have now expanded the Discussion section to address the complexity between connectivity measures and mathematical cognition. The Discussion now states: “Some previous studies in children showed a detrimental influence of stronger functional connectivity in participants with lower mathematical abilities (61, 62). However, the relationship between functional connectivity and math performance is more complex than originally thought and it is influenced by several factors including developmental stage (children vs adults), concurrent maths measures vs learning studies, and in response to interventions (e.g., before vs after maths tutoring). For example, greater IPS connectivity with the premotor cortex and supramarginal gyrus has been associated with better performance in adulthood (63), but worse performance in children (64). Moreover, frontohippocampal connectivity was shown to be negatively correlated with concurrent math measures in adolescence (64), which contrasts with results from learning studies. For example, tutoring-induced strengthened connectivity between IPS with lateral prefrontal cortex (as well as ventral temporal-occipital cortex, and hippocampus) was also associated with improved mathematical in children (65). In previous studies, we have that the association between different brain measures (e.g., GABA, glutamate, aperiodic exponent) and cognition (e.g., maths) can be reversed as a function of the developmental stage (28, 66). Future studies should examine whether and how the different brain indices (GABA, glutamate, functional connectivity, aperiodic exponent) are linked to each other and may reflect the changes in the association between functional connectivity and maths as a function of the developmental stage. (e.g., developmental stage, learning vs performance).” (line=23, paragraph=3, page=21)

**R3.C4.** *Perhaps the training data could also provide further information regarding the complex 4-fold interaction of GABA levels. Depending on the initial GABA level/functional connectivity, the strongest training improvement might be reached earlier than after 5 training sessions (steepness of the performance improvement during training, e.g. in accuracy data) or has not yet been reached at all in other participants. At the very least, this would be interesting to see in the SM*.

**R3.R4.** We thank the reviewer for this suggestion. We have looked into such interesting effects, but the statistical analysis did not support this interesting idea. We have now provided the statistical results for such analysis at the accuracy level that we now present in **Supporting Information 15**. As in our original study (8), there was no effect on accuracy. This can be explained by our paradigm that emphasises accuracy, therefore increasing the sensitivity to detect reaction times effect (60). We have pasted **Supporting Information 15** below.

**Supporting Information 15.** A table depicting the statistical results of the linear mixed-effects model predicting accuracy based on learning type (drill, calculation), tRNS condition (sham tRNS which is the reference group here, dlPFC-tRNS), dlPFC GABA concentration, day, and right frontoparietal connectivity. Statistics: Value=regression coefficient, SE=standard error, DF=degrees of freedom, T=T-value, P=P-value. Interactor predictors are denoted by the * symbol. Neurochemicals: dlPFC= dorsolateral prefrontal cortex, GABA= gamma-aminobutyric acid. A “Δ” prefix denotes a post-tRNS minus pre-tRNS difference score in that neurochemical or functional connectivity measure.

|  | **Value** | **SE** | **DF** | **T** | **P** | **CI_L** | **CI_U** |
| --- | --- | --- | --- | --- | --- | --- | --- |
| (Intercept) | 0.95 | 0.03 | 120 | 32.15 | 0.00 | 0.89 | 1.01 |
| Day | 0.00 | 0.01 | 120 | 0.27 | 0.79 | -0.01 | 0.02 |
| TypeDrill | 0.03 | 0.03 | 120 | 1.02 | 0.31 | -0.03 | 0.10 |
| Δ FC | -0.01 | 0.06 | 8 | -0.23 | 0.82 | -0.16 | 0.13 |
| Δ dlPFC GABA | 0.19 | 0.18 | 8 | 1.02 | 0.34 | -0.24 | 0.61 |
| DlPFC-tRNS | -0.02 | 0.03 | 8 | -0.53 | 0.61 | -0.09 | 0.06 |
| Day*TypeDrill | 0.00 | 0.01 | 120 | 0.05 | 0.96 | -0.02 | 0.02 |
| Day*Δ FC | 0.01 | 0.01 | 120 | 0.52 | 0.61 | -0.02 | 0.04 |
| TypeDrill*Δ FC | 0.06 | 0.07 | 120 | 0.86 | 0.39 | -0.08 | 0.20 |
| Day*Δ dlPFC GABA | -0.01 | 0.04 | 120 | -0.30 | 0.77 | -0.10 | 0.07 |
| TypeDrill*Δ dlPFC GABA | -0.21 | 0.20 | 120 | -1.05 | 0.29 | -0.61 | 0.19 |
| Δ FC*Δ dlPFC GABA | 0.13 | 0.51 | 8 | 0.25 | 0.81 | -1.05 | 1.30 |
| Day*DlPFC-tRNS | 0.00 | 0.01 | 120 | 0.53 | 0.60 | -0.01 | 0.02 |
| TypeDrill*DlPFC-tRNS | 0.03 | 0.04 | 120 | 0.79 | 0.43 | -0.04 | 0.10 |
| Δ FC*DlPFC-tRNS | -0.02 | 0.08 | 8 | -0.25 | 0.81 | -0.21 | 0.17 |
| Δ dlPFC GABA*DlPFC-tRNS | -0.17 | 0.19 | 8 | -0.88 | 0.40 | -0.62 | 0.28 |
| Day*TypeDrill*Δ FC | -0.03 | 0.02 | 120 | -1.20 | 0.23 | -0.07 | 0.02 |
| Day*TypeDrill*Δ dlPFC GABA | 0.06 | 0.06 | 120 | 0.95 | 0.34 | -0.06 | 0.18 |
| Day*Δ FC*Δ dlPFC GABA | -0.09 | 0.12 | 120 | -0.74 | 0.46 | -0.33 | 0.15 |
| TypeDrill*Δ FC*Δ dlPFC GABA | -0.05 | 0.56 | 120 | -0.09 | 0.93 | -1.17 | 1.06 |
| Day*TypeDrill*DlPFC-tRNS | -0.02 | 0.01 | 120 | -1.40 | 0.16 | -0.04 | 0.01 |
| Day*Δ FC*DlPFC-tRNS | -0.01 | 0.02 | 120 | -0.42 | 0.67 | -0.05 | 0.03 |
| TypeDrill*Δ FC*DlPFC-tRNS | -0.02 | 0.09 | 120 | -0.18 | 0.86 | -0.19 | 0.16 |
| Day*Δ dlPFC GABA*DlPFC-tRNS | 0.02 | 0.05 | 120 | 0.40 | 0.69 | -0.07 | 0.11 |
| TypeDrill*Δ dlPFC GABA*DlPFC-tRNS | 0.24 | 0.21 | 120 | 1.12 | 0.27 | -0.19 | 0.66 |
| Δ FC*Δ dlPFC GABA*DlPFC-tRNS | -0.36 | 0.52 | 8 | -0.68 | 0.52 | -1.56 | 0.85 |
| Day*TypeDrill*Δ FC*Δ dlPFC GABA | -0.02 | 0.17 | 120 | -0.09 | 0.93 | -0.35 | 0.32 |
| Day*TypeDrill*Δ FC*DlPFC-tRNS | 0.01 | 0.03 | 120 | 0.50 | 0.62 | -0.04 | 0.07 |
| Day*TypeDrill*Δ dlPFC GABA*DlPFC-tRNS | -0.08 | 0.06 | 120 | -1.18 | 0.24 | -0.20 | 0.05 |
| Day*Δ FC*Δ dlPFC GABA*DlPFC-tRNS | 0.11 | 0.12 | 120 | 0.86 | 0.39 | -0.14 | 0.35 |
| TypeDrill*Δ FC*Δ dlPFC GABA*DlPFC-tRNS | 0.28 | 0.58 | 120 | 0.48 | 0.63 | -0.87 | 1.42 |
| Day*TypeDrill*Δ FC*Δ dlPFC GABA*DlPFC-tRNS | -0.06 | 0.17 | 120 | -0.32 | 0.75 | -0.40 | 0.29 |

**R3.C5.** *At the beginning of the methods section, it should be made explicit and clear to readers that participants were divided into three groups, and demographic data should be given for these three groups (this information is currently provided in the SM). While the grouping becomes clearer later in the manuscript, at this point, it initially gave the impression of a within-subject design. Of course, given the potential for long-term effects, a within-subject design would not be feasible, but it is important to clarify from the outset that this study examines between-subject effects, along with all the associated limitations.*

**R3.R5.** We have now made explicit and clear to readers at the beginning of the methods section that participants were randomly assigned to three different groups, and demographic data should be given for these three groups. The text now states: “We recruited 72 healthy, right-handed participants (36 males, mean age=21.75, SD=3.21, range=18-30), and 36 females (mean age=22.1, SD=2.63, range=19-28). Participants were randomly assigned into three stimulation groups (i.e., sham tRNS vs. active dlPFC-tRNS vs. active PPC-tRNS), and each of the three stimulation groups consisted of 12 females and 12 males.” (line=7, paragraph=1,page=8)

**R3.C6.** *The results of GABAergic modulation are not clearly stated in the abstract.*

**R3.R6.** We have now clearly stated in the abstract the GABAergic modulation results. The text now reads: “Further analyses revealed that dlPFC-tRNS improved learning outcomes for participants who showed reductions in dlPFC GABA when it was accompanied by a reduced positive frontoparietal functional connectivity but this effect was reversed for participants who showed increased positive frontoparietal functional connectivity.” (line=20, paragraph=1, page=2)

**R3.C7.** *Instead of an introduction, the manuscript has a summary, probably a typo*

**R3.R7.** We thank the reviewer for noticing this, we have now replaced the word “Summary” with “Introduction”. (line=5, paragraph=2, page=3)

**R3.C8.** *There should also be references for the Matthew effect in the introduction (p. 3), not just in the discussion.*

**R3.R8.** We have now added references for the Matthew effect in the introduction. The text now states: “However, not all individuals benefit equally from educational opportunities, a phenomenon referred to as the Matthew effect (52) in education.” (line=7, paragraph=2, page=3)

**R3.C9.** *p. 3: „Longitudinal studies indicate that mathematical abilities are relatively stable from childhood through adulthood, driven primarily by biological rather than environmental factors (6-8)." But there are also alternative suggestions, according to Judd et al. (2020; PNAS), this is independently influenced by socioeconomic status and polygenetic factors.*

**R3.R9.** We thank the reviewer for the suggestion and have also added these alternative suggestions. The text now states: "Our results, in line with previous results (56-58), support the view that such differences may stem from biological origin. However, such differences may be independently influenced by socioeconomic status and polygenetic factors (59). By using a learning task and modulating the neural correlate involved in mathematical learning we were able to show that the Matthews effect can be explained and mitigated at the biological level. Our results do not undermine the environmental factors (e.g., socioeconomic status) that could also draw a causal effect but are less likely to play a role in this study due to the random allocation of participants, and using a learning task. Our results highlight the possibility of reversing such biological disadvantage using an excitatory form of neurostimulation to target the neural substrates involved in calculation learning. This is in line with a recent study that identified genes that correlate with mathematical abilities, as crucial for neuronal excitability (58). Future studies can build on our findings by assessing how long this effect lasts, and whether such an approach can have practical relevance outside the controlled lab environment. Answering these questions would have important implications for the fields of education and science of learning.” (line=14, paragraph=2, page=17).

**R3.C10.** *p. 4: "…mathematical learning by modulating brain activity and neurochemicals involved…". -> "…mathematical learning by cortical excitability and neurochemicals involved… (Nitsche et al., 2005)".*

**R3.R10.** We have now amended the text accordingly. The text now reads: “Non-invasive neurostimulation techniques offer a promising avenue for exploring the causal mechanisms of mathematical learning by modulating cortical excitability and neurochemicals involved in cognitive tasks (67-69).” (line=3, paragraph=2, page=4)

**R3.C11.** *P. 5: "tRNS, due to its suggested excitatory effect is hypothesized to enhance plasticity later in life". Again, this sounds as if it has been proven and recognized that a permanent change in neuro-cognitive plasticity can be achieved over a lifetime by using tRNS in 5 sessions. If there is a study that has shown this, please cite it here (and elsewhere), otherwise such statements do not belong here.*

**R3.R11.** We clarified now the sentence and wrote "tRNS, due to its potential excitatory effect, has been suggested to enhance plasticity with a continuous effect beyond the stimulation session, as indicated by lasting effects beyond the stimulation period (e.g., weeks to months; (8, 9, 17, 23-27)." (line=18, paragraph=2, page=6)

**R3.C12.** *P. 8: "A scan was performed…" -> "A scan* *(i.e., H-MRS and resting state fMRI) was performed…"*

**R3.R12.** We have now amended the text accordingly. The text now reads: “A scan (i.e., structural MRI, ^1^H-MRS and resting state fMRI).” (line=4, paragraph=1, page=10)

**R3.C13.** *In my opinion, the behavioral data results should be included in the main manuscript, along with a table in the SM containing descriptive statistics of the behavioral data. These are essential for readers to evaluate the magnitude of the manipulations.*

**R3.R13.** Done. We have now provided the behavioural results in Table 1 and **Supporting Information 19,** and the descriptive statistics in **Supporting Information 17**, all of which are pasted below.

**Table 1**. Behavioural results when predicting reaction time from stimulation condition (compared to sham tRNS, which serves as the reference), day and type. Statistics: Value=regression coefficient, SE=standard error, DF=degrees of freedom, T=t-value, P=p-value, dlPFC=dorsolateral prefrontal cortex, PPC=posterior parietal cortex. Interactions are denoted by the * symbol.

|  | **Value** | **SE** | **DF** | **T** | **P** | **CI_L** | **CI_U** |
| --- | --- | --- | --- | --- | --- | --- | --- |
| (Intercept) | 3152.746 | 150.463 | 639 | 20.954 | <.00001 | 2857.283 | 3448.209 |
| TypeDrill | -2373.140 | 166.636 | 639 | -14.241 | <.00001 | -2700.360 | -2045.920 |
| Day | -297.111 | 35.527 | 639 | -8.363 | <.00001 | -366.875 | -227.347 |
| dlPFC-tRNS | -535.643 | 196.507 | 69 | -2.726 | 0.0081 | -927.664 | -143.622 |
| PPC-tRNS | -343.411 | 209.225 | 69 | -1.641 | 0.1053 | -760.804 | 73.983 |
| TypeDrill*Day | 252.765 | 50.243 | 639 | 5.031 | <.00001 | 154.104 | 351.425 |
| TypeDrill*dlPFC-tRNS | 543.069 | 205.446 | 639 | 2.643 | 0.0084 | 139.638 | 946.499 |
| TypeDrill*dlPFC-PPC | 260.252 | 229.191 | 639 | 1.136 | 0.2566 | -189.807 | 710.310 |
| Day*dlPFC-tRNS | 82.866 | 43.801 | 639 | 1.892 | 0.0590 | -3.146 | 168.878 |
| Day*PPC-tRNS | 51.874 | 48.864 | 639 | 1.062 | 0.2888 | -44.079 | 147.827 |
| TypeDrill*Day*dlPFC-tRNS | -94.401 | 61.944 | 639 | -1.524 | 0.1280 | -216.040 | 27.238 |
| TypeDrill*Day*PPC-tRNS | -45.750 | 69.104 | 639 | -0.662 | 0.5082 | -181.448 | 89.947 |

**Supporting Information 19.** Behavioural results when predicting accuracy from stimulation condition (compared to sham tRNS, which serves as the reference), day and type. Statistics: Value=regression coefficient, SE=standard error, DF=degrees of freedom, T=t-value, P=p-value, dlPFC=dorsolateral prefrontal cortex, PPC=posterior parietal cortex. Interactions are denoted by the * symbol.

|  | **Value** | **SE** | **DF** | **T** | **P** | **CI_L** | **CI_U** |
| --- | --- | --- | --- | --- | --- | --- | --- |
| (Intercept) | 0.922 | 0.010 | 639 | 88.361 | <.00001 | 0.902 | 0.943 |
| TypeDrill | 0.063 | 0.011 | 639 | 5.696 | <.00001 | 0.041 | 0.084 |
| Day | 0.002 | 0.002 | 639 | 0.686 | 0.4932 | -0.003 | 0.006 |
| dlPFC-tRNS | -0.001 | 0.014 | 69 | -0.050 | 0.9602 | -0.028 | 0.027 |
| PPC-tRNS | -0.002 | 0.017 | 69 | -0.101 | 0.9200 | -0.035 | 0.031 |
| TypeDrill*Day | -0.012 | 0.003 | 639 | -3.599 | 0.0003 | -0.018 | -0.005 |
| TypeDrill*dlPFC-tRNS | -0.015 | 0.014 | 639 | -1.083 | 0.2793 | -0.043 | 0.012 |
| TypeDrill*PPC-tRNS | -0.014 | 0.019 | 639 | -0.759 | 0.4479 | -0.051 | 0.023 |
| Day*dlPFC-tRNS | 0.003 | 0.003 | 639 | 1.171 | 0.2419 | -0.002 | 0.009 |
| Day*PPC-tRNS | -0.003 | 0.004 | 639 | -0.671 | 0.5022 | -0.011 | 0.005 |
| TypeDrill*Day*dlPFC-tRNS | 0.001 | 0.004 | 639 | 0.225 | 0.8218 | -0.007 | 0.009 |
| TypeDrill*Day*PPC-tRNS | 0.003 | 0.006 | 639 | 0.485 | 0.6278 | -0.008 | 0.014 |

**Supporting Information 17.** Mean and standard deviation (SD) of reaction time and accuracy separately for learning type (Drill, Calculation), stimulation groups (Sham-tRNS, dlPFC-tRNS and PPC-tRNS), day (1 to 5), and block.

|  | **Sham-tRNS** | | **dlPFC-tRNS** | | **PPC-tRNS** | |
| --- | --- | --- | --- | --- | --- | --- |
|  | **Mean** | **SD** | **Mean** | **SD** | **Mean** | **SD** |
| Drill RT Day 1 Block 1 | 902.67 | 304.85 | 973.53 | 404.03 | 810.95 | 385.84 |
| Drill RT Day 1 Block 2 | 809.32 | 266.04 | 788.87 | 279.58 | 745.60 | 370.08 |
| Drill RT Day 1 Block 3 | 710.17 | 224.93 | 718.21 | 277.99 | 676.39 | 389.44 |
| Drill RT Day 1 Block 4 | 722.44 | 250.27 | 665.72 | 270.95 | 676.35 | 378.87 |
| Drill RT Day 1 Block 5 | 675.91 | 254.74 | 646.54 | 249.55 | 727.29 | 356.28 |
| Drill RT Day 2 Block 1 | 820.03 | 328.62 | 735.56 | 336.07 | 689.60 | 335.65 |
| Drill RT Day 2 Block 2 | 721.15 | 301.38 | 711.63 | 356.67 | 621.06 | 276.61 |
| Drill RT Day 2 Block 3 | 700.75 | 284.57 | 656.41 | 328.73 | 576.77 | 258.47 |
| Drill RT Day 2 Block 4 | 686.87 | 281.11 | 661.63 | 298.31 | 549.99 | 257.44 |
| Drill RT Day 2 Block 5 | 632.59 | 256.60 | 667.80 | 334.35 | 547.00 | 273.86 |
| Drill RT Day 2 Block 6 | 629.17 | 231.41 | 680.85 | 353.71 | 529.44 | 205.73 |
| Drill RT Day 3 Block 1 | 670.32 | 281.09 | 648.72 | 286.20 | 590.00 | 235.38 |
| Drill RT Day 3 Block 2 | 647.71 | 267.86 | 586.72 | 248.28 | 522.13 | 213.04 |
| Drill RT Day 3 Block 3 | 592.92 | 230.55 | 564.22 | 258.47 | 546.99 | 249.57 |
| Drill RT Day 3 Block 4 | 591.18 | 226.84 | 549.74 | 231.43 | 503.68 | 206.98 |
| Drill RT Day 3 Block 5 | 598.57 | 276.38 | 535.64 | 254.15 | 474.42 | 183.37 |
| Drill RT Day 3 Block 6 | 589.53 | 232.97 | 550.96 | 270.75 | 487.82 | 213.51 |
| Drill RT Day 3 Block 7 | 547.02 | 225.18 | 537.07 | 212.53 | 481.00 | 194.76 |
| Drill RT Day 4 Block 1 | 650.57 | 215.47 | 577.43 | 187.06 | 560.27 | 187.56 |
| Drill RT Day 4 Block 2 | 590.89 | 207.31 | 556.96 | 205.12 | 501.91 | 199.02 |
| Drill RT Day 4 Block 3 | 544.46 | 186.24 | 532.98 | 175.92 | 514.28 | 196.40 |
| Drill RT Day 4 Block 4 | 530.11 | 210.41 | 519.51 | 175.90 | 492.47 | 200.65 |
| Drill RT Day 4 Block 5 | 514.04 | 199.87 | 513.14 | 189.01 | 497.25 | 216.28 |
| Drill RT Day 4 Block 6 | 521.69 | 204.92 | 522.13 | 214.94 | 512.74 | 250.85 |
| Drill RT Day 4 Block 7 | 505.95 | 196.25 | 491.85 | 174.61 | 480.55 | 199.07 |
| Drill RT Day 4 Block 8 | 516.63 | 203.32 | 496.37 | 177.21 | 468.76 | 187.46 |
| Drill RT Day 5 Block 1 | 728.08 | 208.88 | 623.92 | 213.22 | 665.82 | 235.39 |
| Drill RT Day 5 Block 2 | 642.13 | 201.47 | 577.26 | 205.38 | 603.79 | 208.60 |
| Drill RT Day 5 Block 3 | 625.39 | 218.69 | 562.43 | 237.26 | 566.54 | 236.11 |
| Drill RT Day 5 Block 4 | 613.26 | 196.43 | 562.42 | 202.54 | 567.47 | 242.13 |
| Drill RT Day 5 Block 5 | 583.75 | 208.46 | 546.47 | 210.24 | 533.75 | 223.58 |
| Drill RT Day 5 Block 6 | 570.95 | 198.55 | 512.45 | 199.28 | 564.06 | 240.49 |
| Drill RT Day 5 Block 7 | 563.78 | 213.43 | 526.97 | 183.17 | 539.38 | 242.65 |
| Calculation RT Day 1 Block 1 | 4102.23 | 1885.26 | 3785.48 | 1824.52 | 3773.01 | 1871.60 |
| Calculation RT Day 1 Block 2 | 3266.25 | 1530.21 | 2616.45 | 1178.83 | 2947.57 | 1583.46 |
| Calculation RT Day 1 Block 3 | 3058.15 | 1214.40 | 2477.21 | 1140.13 | 2805.17 | 1419.94 |
| Calculation RT Day 1 Block 4 | 2688.55 | 1196.16 | 2203.18 | 819.50 | 2347.05 | 1134.50 |
| Calculation RT Day 1 Block 5 | 2489.77 | 1199.64 | 2144.43 | 810.72 | 2297.03 | 1173.12 |
| Calculation RT Day 2 Block 1 | 2871.76 | 1379.71 | 2281.99 | 865.73 | 2600.58 | 1353.24 |
| Calculation RT Day 2 Block 2 | 2433.37 | 1096.62 | 2185.16 | 997.84 | 2094.46 | 1089.58 |
| Calculation RT Day 2 Block 3 | 2481.73 | 1185.74 | 2045.35 | 716.15 | 2093.59 | 1083.94 |
| Calculation RT Day 2 Block 4 | 2299.56 | 952.06 | 1947.41 | 679.16 | 2085.75 | 966.15 |
| Calculation RT Day 2 Block 5 | 2056.18 | 908.67 | 1896.43 | 769.86 | 1940.78 | 912.14 |
| Calculation RT Day 2 Block 6 | 1949.51 | 934.42 | 1869.15 | 632.62 | 1841.44 | 869.63 |
| Calculation RT Day 3 Block 1 | 2234.42 | 851.55 | 1995.87 | 751.50 | 1949.83 | 741.64 |
| Calculation RT Day 3 Block 2 | 2311.68 | 1053.28 | 1848.85 | 632.12 | 1863.39 | 742.69 |
| Calculation RT Day 3 Block 3 | 2116.92 | 949.46 | 1770.64 | 585.08 | 1832.37 | 814.48 |
| Calculation RT Day 3 Block 4 | 2092.09 | 1162.40 | 1744.52 | 566.24 | 1956.05 | 831.50 |
| Calculation RT Day 3 Block 5 | 1962.64 | 1114.22 | 1704.03 | 597.54 | 1878.57 | 828.80 |
| Calculation RT Day 3 Block 6 | 2019.76 | 1051.91 | 1688.22 | 611.40 | 1787.63 | 778.31 |
| Calculation RT Day 3 Block 7 | 1868.57 | 985.01 | 1618.73 | 620.71 | 1844.39 | 897.82 |
| Calculation RT Day 4 Block 1 | 2019.62 | 739.90 | 1671.43 | 515.80 | 1807.79 | 704.38 |
| Calculation RT Day 4 Block 2 | 1875.84 | 702.41 | 1588.18 | 480.57 | 1787.59 | 857.73 |
| Calculation RT Day 4 Block 3 | 1911.42 | 883.38 | 1637.59 | 504.20 | 1748.96 | 718.07 |
| Calculation RT Day 4 Block 4 | 1992.85 | 986.21 | 1782.84 | 780.31 | 1880.85 | 839.67 |
| Calculation RT Day 4 Block 5 | 1766.88 | 845.68 | 1709.75 | 690.57 | 1685.95 | 740.40 |
| Calculation RT Day 4 Block 6 | 1948.99 | 1009.91 | 1643.32 | 635.87 | 1882.42 | 946.75 |
| Calculation RT Day 4 Block 7 | 1804.06 | 843.55 | 1525.88 | 580.97 | 1716.46 | 907.25 |
| Calculation RT Day 4 Block 8 | 1736.82 | 671.49 | 1700.63 | 662.69 | 1714.79 | 882.92 |
| Calculation RT Day 5 Block 1 | 2039.61 | 1022.35 | 1913.01 | 891.64 | 1844.42 | 727.91 |
| Calculation RT Day 5 Block 2 | 1868.56 | 844.75 | 1763.75 | 741.07 | 1870.83 | 993.41 |
| Calculation RT Day 5 Block 3 | 1768.47 | 755.72 | 1751.51 | 614.78 | 1803.08 | 846.90 |
| Calculation RT Day 5 Block 4 | 1842.66 | 868.69 | 1846.78 | 783.46 | 1717.31 | 687.59 |
| Calculation RT Day 5 Block 5 | 1851.62 | 901.66 | 1731.57 | 651.50 | 1728.77 | 721.23 |
| Calculation RT Day 5 Block 6 | 1852.34 | 934.22 | 1681.16 | 667.63 | 1748.83 | 783.71 |
| Calculation RT Day 5 Block 7 | 1857.98 | 986.17 | 1661.54 | 683.29 | 1700.89 | 763.51 |
| Drill Accuracy Day 1 Block 1 | 0.95 | 0.06 | 0.95 | 0.05 | 0.92 | 0.08 |
| Drill Accuracy Day 1 Block 2 | 0.97 | 0.04 | 0.97 | 0.04 | 0.93 | 0.07 |
| Drill Accuracy Day 1 Block 3 | 0.96 | 0.04 | 0.96 | 0.05 | 0.95 | 0.06 |
| Drill Accuracy Day 1 Block 4 | 0.95 | 0.05 | 0.95 | 0.05 | 0.95 | 0.06 |
| Drill Accuracy Day 1 Block 5 | 0.96 | 0.05 | 0.96 | 0.06 | 0.94 | 0.09 |
| Drill Accuracy Day 2 Block 1 | 0.96 | 0.06 | 0.96 | 0.03 | 0.94 | 0.06 |
| Drill Accuracy Day 2 Block 2 | 0.97 | 0.04 | 0.95 | 0.06 | 0.94 | 0.08 |
| Drill Accuracy Day 2 Block 3 | 0.99 | 0.03 | 0.96 | 0.05 | 0.95 | 0.06 |
| Drill Accuracy Day 2 Block 4 | 0.96 | 0.04 | 0.94 | 0.06 | 0.98 | 0.02 |
| Drill Accuracy Day 2 Block 5 | 0.98 | 0.04 | 0.94 | 0.07 | 0.96 | 0.06 |
| Drill Accuracy Day 2 Block 6 | 0.98 | 0.03 | 0.98 | 0.04 | 0.97 | 0.05 |
| Drill Accuracy Day 3 Block 1 | 0.97 | 0.06 | 0.95 | 0.10 | 0.94 | 0.13 |
| Drill Accuracy Day 3 Block 2 | 0.96 | 0.03 | 0.94 | 0.07 | 0.95 | 0.07 |
| Drill Accuracy Day 3 Block 3 | 0.96 | 0.05 | 0.98 | 0.05 | 0.96 | 0.05 |
| Drill Accuracy Day 3 Block 4 | 0.96 | 0.05 | 0.97 | 0.04 | 0.97 | 0.04 |
| Drill Accuracy Day 3 Block 5 | 0.96 | 0.06 | 0.96 | 0.04 | 0.96 | 0.06 |
| Drill Accuracy Day 3 Block 6 | 0.97 | 0.05 | 0.97 | 0.05 | 0.96 | 0.05 |
| Drill Accuracy Day 3 Block 7 | 0.98 | 0.04 | 0.97 | 0.04 | 0.96 | 0.05 |
| Drill Accuracy Day 4 Block 1 | 0.94 | 0.07 | 0.94 | 0.09 | 0.95 | 0.09 |
| Drill Accuracy Day 4 Block 2 | 0.96 | 0.05 | 0.95 | 0.07 | 0.94 | 0.12 |
| Drill Accuracy Day 4 Block 3 | 0.95 | 0.07 | 0.97 | 0.05 | 0.98 | 0.04 |
| Drill Accuracy Day 4 Block 4 | 0.96 | 0.05 | 0.97 | 0.04 | 0.95 | 0.07 |
| Drill Accuracy Day 4 Block 5 | 0.95 | 0.07 | 0.97 | 0.06 | 0.96 | 0.04 |
| Drill Accuracy Day 4 Block 6 | 0.96 | 0.04 | 0.96 | 0.05 | 0.96 | 0.04 |
| Drill Accuracy Day 4 Block 7 | 0.95 | 0.07 | 0.96 | 0.05 | 0.94 | 0.05 |
| Drill Accuracy Day 4 Block 8 | 0.94 | 0.07 | 0.94 | 0.06 | 0.95 | 0.06 |
| Drill Accuracy Day 5 Block 1 | 0.86 | 0.16 | 0.90 | 0.08 | 0.87 | 0.18 |
| Drill Accuracy Day 5 Block 2 | 0.93 | 0.08 | 0.91 | 0.09 | 0.86 | 0.17 |
| Drill Accuracy Day 5 Block 3 | 0.93 | 0.07 | 0.94 | 0.07 | 0.90 | 0.11 |
| Drill Accuracy Day 5 Block 4 | 0.94 | 0.06 | 0.93 | 0.08 | 0.88 | 0.14 |
| Drill Accuracy Day 5 Block 5 | 0.92 | 0.07 | 0.94 | 0.08 | 0.91 | 0.10 |
| Drill Accuracy Day 5 Block 6 | 0.91 | 0.08 | 0.94 | 0.07 | 0.89 | 0.10 |
| Drill Accuracy Day 5 Block 7 | 0.93 | 0.09 | 0.92 | 0.06 | 0.90 | 0.12 |
| Calculation Accuracy Day 1 Block 1 | 0.92 | 0.08 | 0.91 | 0.07 | 0.91 | 0.09 |
| Calculation Accuracy Day 1 Block 2 | 0.94 | 0.05 | 0.94 | 0.06 | 0.95 | 0.06 |
| Calculation Accuracy Day 1 Block 3 | 0.92 | 0.08 | 0.93 | 0.06 | 0.92 | 0.06 |
| Calculation Accuracy Day 1 Block 4 | 0.94 | 0.05 | 0.94 | 0.08 | 0.93 | 0.09 |
| Calculation Accuracy Day 1 Block 5 | 0.93 | 0.06 | 0.92 | 0.08 | 0.91 | 0.08 |
| Calculation Accuracy Day 2 Block 1 | 0.92 | 0.08 | 0.95 | 0.06 | 0.89 | 0.08 |
| Calculation Accuracy Day 2 Block 2 | 0.90 | 0.06 | 0.93 | 0.10 | 0.91 | 0.08 |
| Calculation Accuracy Day 2 Block 3 | 0.95 | 0.08 | 0.94 | 0.06 | 0.93 | 0.06 |
| Calculation Accuracy Day 2 Block 4 | 0.93 | 0.07 | 0.92 | 0.08 | 0.90 | 0.12 |
| Calculation Accuracy Day 2 Block 5 | 0.93 | 0.06 | 0.92 | 0.13 | 0.90 | 0.08 |
| Calculation Accuracy Day 2 Block 6 | 0.93 | 0.07 | 0.93 | 0.07 | 0.90 | 0.09 |
| Calculation Accuracy Day 3 Block 1 | 0.89 | 0.08 | 0.93 | 0.06 | 0.91 | 0.08 |
| Calculation Accuracy Day 3 Block 2 | 0.90 | 0.17 | 0.94 | 0.06 | 0.92 | 0.07 |
| Calculation Accuracy Day 3 Block 3 | 0.95 | 0.05 | 0.93 | 0.07 | 0.92 | 0.08 |
| Calculation Accuracy Day 3 Block 4 | 0.92 | 0.05 | 0.93 | 0.08 | 0.92 | 0.10 |
| Calculation Accuracy Day 3 Block 5 | 0.93 | 0.07 | 0.96 | 0.06 | 0.95 | 0.06 |
| Calculation Accuracy Day 3 Block 6 | 0.95 | 0.05 | 0.94 | 0.07 | 0.94 | 0.07 |
| Calculation Accuracy Day 3 Block 7 | 0.92 | 0.09 | 0.96 | 0.06 | 0.91 | 0.07 |
| Calculation Accuracy Day 4 Block 1 | 0.92 | 0.07 | 0.94 | 0.08 | 0.92 | 0.06 |
| Calculation Accuracy Day 4 Block 2 | 0.92 | 0.08 | 0.92 | 0.08 | 0.91 | 0.07 |
| Calculation Accuracy Day 4 Block 3 | 0.94 | 0.05 | 0.92 | 0.08 | 0.92 | 0.06 |
| Calculation Accuracy Day 4 Block 4 | 0.93 | 0.07 | 0.91 | 0.11 | 0.89 | 0.14 |
| Calculation Accuracy Day 4 Block 5 | 0.94 | 0.06 | 0.95 | 0.06 | 0.91 | 0.08 |
| Calculation Accuracy Day 4 Block 6 | 0.93 | 0.07 | 0.94 | 0.06 | 0.93 | 0.06 |
| Calculation Accuracy Day 4 Block 7 | 0.94 | 0.06 | 0.95 | 0.07 | 0.91 | 0.08 |
| Calculation Accuracy Day 4 Block 8 | 0.93 | 0.09 | 0.94 | 0.07 | 0.92 | 0.09 |
| Calculation Accuracy Day 5 Block 1 | 0.94 | 0.05 | 0.95 | 0.06 | 0.87 | 0.16 |
| Calculation Accuracy Day 5 Block 2 | 0.91 | 0.12 | 0.93 | 0.06 | 0.90 | 0.09 |
| Calculation Accuracy Day 5 Block 3 | 0.95 | 0.07 | 0.96 | 0.06 | 0.91 | 0.11 |
| Calculation Accuracy Day 5 Block 4 | 0.94 | 0.07 | 0.96 | 0.05 | 0.93 | 0.07 |
| Calculation Accuracy Day 5 Block 5 | 0.94 | 0.06 | 0.96 | 0.05 | 0.93 | 0.08 |
| Calculation Accuracy Day 5 Block 6 | 0.92 | 0.07 | 0.95 | 0.05 | 0.92 | 0.07 |
| Calculation Accuracy Day 5 Block 7 | 0.92 | 0.08 | 0.96 | 0.06 | 0.94 | 0.05 |

**R3.C14.** *Figure 2 is a bit confusing as in panels B and C the colours indicate the values for mean and SDs, while in D and E they indicate stimulation type. Would it be possible to make the layout of the illustrations more uniform or at least use different colors?*

**R3.R14.** Yes, certainly, we have now used different colours in **Fig. 2**, which we now paste below.

**R3.C15.** *P. 14: "In this study, we examined how tRNS can alter mathematical learning…" Again, due to the lack of a non-mathematical control task this is hard to defend.*

**R3.R15.** We would like to take the opportunity and clarify as in R3.C1 that this sentence does not mean that it only alters mathematical learning. We can still examine how tRNS can alter mathematical learning, but this can occur via changes that are domain-specific or general. This however was not the aim of this project. We have now clarified this issue in the Discussion, which states, “This study provides novel insights into the neural mechanisms underlying mathematical learning, specifically in terms of the role of dlPFC in calculation but not drill learning in adults. Namely, in contrast to previous fMRI studies that highlighted the role of dlPFC also in drill learning, our findings suggest that dlPFC may instead be a correlate and may indicate redundancy in brain functions (29) with regards to drill learning in the adult brain. While we included a mathematical control task, future studies should include a non-mathematical learning control task that also heavily depends on executive functions to help distinguish between specific executive function components. This will allow the examination of whether the observed neurostimulation effect is specific to mathematical learning or due to a domain-general modulation of executive functions. Nevertheless, our findings are theoretically consistent with current meta-analyses and models of arithmetic processing (6, 10, 30-34). Our two-time point (i.e., before vs after tRNS) additional analyses did not provide evidence that tRNS modulated working memory/executive functions (**Supporting Information 16**). Recent tRNS experiments that used a similar prefrontal montage as in this study, improved sustained attention performance or symptoms that are associated with sustained attention impairments (17, 24, 35). Therefore, we hypothesize that our results are due to sustained attention enhancement that has been linked with improved academic learning (36).” (line=22, paragraph=2, page=22)

In summary, we would like to thank you again for the constructive feedback and for inviting us to submit this revision. We are pleased with the revised manuscript and look forward to hearing from you about our submission.

Sincerely,

Dr George Zacharopoulos *et al.*

**References**

1.	T. Yarkoni, R. A. Poldrack, T. E. Nichols, D. C. Van Essen, T. D. Wager, Large-scale automated synthesis of human functional neuroimaging data. *Nature methods* **8**, 665-670 (2011).

2.	V. Menon, "Arithmetic in the child and adult brain" in The Oxford handbook of numerical cognition*,* R. Cohen Kadosh, A. Dowker, Eds. (Oxford University Press, 2015), pp. 502–530.

3.	M. G. Von Aster, R. S. Shalev, Number development and developmental dyscalculia. *Developmental medicine & child neurology* **49**, 868-873 (2007).

4.	J. M. Chein, W. Schneider, The brain’s learning and control architecture. *Current directions in psychological science* **21**, 78-84 (2012).

5.	L. Cragg, C. Gilmore, Skills underlying mathematics: The role of executive function in the development of mathematics proficiency. *Trends in neuroscience and education* **3**, 63-68 (2014).

6.	M. Arsalidou, M. J. Taylor, Is 2+ 2= 4? Meta-analyses of brain areas needed for numbers and calculations. *Neuroimage* **54**, 2382-2393 (2011).

7.	L. Zamarian, A. Ischebeck, M. Delazer, Neuroscience of learning arithmetic—Evidence from brain imaging studies. *Neuroscience & Biobehavioral Reviews* **33**, 909-925 (2009).

8.	A. Snowball *et al.*, Long-term enhancement of brain function and cognition using cognitive training and brain stimulation. *Current Biology* **23**, 987-992 (2013).

9.	A. Pasqualotto, B. Kobanbay, M. J. Proulx, Neural Stimulation Has a Long‐Term Effect on Foreign Vocabulary Acquisition. *Neural Plasticity* **2015**, 671705 (2015).

10.	V. Menon, Memory and cognitive control circuits in mathematical cognition and learning. *Progress in brain research* **227**, 159-186 (2016).

11.	S. Qin *et al.*, Hippocampal-neocortical functional reorganization underlies children's cognitive development. *Nature neuroscience* **17**, 1263-1269 (2014).

12.	J. F. Werker, T. K. Hensch, Critical periods in speech perception: new directions. *Annual review of psychology* **66**, 173-196 (2015).

13.	T. K. Hensch, Critical period plasticity in local cortical circuits. *Nature Reviews Neuroscience* **6**, 877-888 (2005).

14.	K. Cohen Kadosh, B. Krause, A. J. King, J. Near, R. Cohen Kadosh, Linking GABA and glutamate levels to cognitive skill acquisition during development. *Human brain mapping* **36**, 4334-4345 (2015).

15.	T. Yamada, T. Watanabe, Y. Sasaki, Plasticity–stability dynamics during post-training processing of learning. *Trends in Cognitive Sciences* (2024).

16.	N. E. van Bueren, S. H. van der Ven, S. Hochman, F. Sella, R. C. Kadosh, Human neuronal excitation/inhibition balance explains and predicts neurostimulation induced learning benefits. *PLoS biology* **21**, e3002193 (2023).

17.	O. Dakwar-Kawar *et al.*, Transcranial random noise stimulation combined with cognitive training for treating ADHD: a randomized, sham-controlled clinical trial. *Translational Psychiatry* **13**, 271 (2023).

18.	C. A. Sánchez-León *et al.*, Impact of chronic transcranial random noise stimulation (tRNS) on GABAergic and glutamatergic activity markers in the prefrontal cortex of juvenile mice. *Progress in brain research* **264**, 323-341 (2021).

19.	L. Chaieb, A. Antal, W. Paulus, Transcranial random noise stimulation-induced plasticity is NMDA-receptor independent but sodium-channel blocker and benzodiazepines sensitive. *Frontiers in neuroscience* **9**, 125 (2015).

20.	G. Zacharopoulos, R. K. Cohen, Predicting working memory capacity based on glutamatergic concentration and its modulation of functional connectivity. *Neuroscience* **457**, 12-19 (2021).

21.	G. Ende, Proton magnetic resonance spectroscopy: relevance of glutamate and GABA to neuropsychology. *Neuropsychology review* **25**, 315-325 (2015).

22.	P. Frangou, M. Correia, Z. Kourtzi, GABA, not BOLD, reveals dissociable learning-dependent plasticity mechanisms in the human brain. *elife* **7**, e35854 (2018).

23.	L. Battelli *et al.*, Delayed long term impact of working memory training coupled with tRNS on near-and far-transfer cognitive tasks. *Journal of Vision* **22**, 4286-4286 (2022).

24.	I. Berger, O. Dakwar-Kawar, E. S. Grossman, M. Nahum, R. C. Kadosh, Scaffolding the attention-deficit/hyperactivity disorder brain using transcranial direct current and random noise stimulation: A randomized controlled trial. *Clinical Neurophysiology* **132**, 699-707 (2021).

25.	C. Brevet-Aeby, M. Mondino, E. Poulet, J. Brunelin, Three repeated sessions of transcranial random noise stimulation (tRNS) leads to long-term effects on reaction time in the Go/No Go task. *Neurophysiologie Clinique* **49**, 27-32 (2019).

26.	M. Cappelletti *et al.*, Transfer of cognitive training across magnitude dimensions achieved with concurrent brain stimulation of the parietal lobe. *Journal of Neuroscience* **33**, 14899-14907 (2013).

27.	F. Herpich *et al.*, Boosting learning efficacy with noninvasive brain stimulation in intact and brain-damaged humans. *Journal of Neuroscience* **39**, 5551-5561 (2019).

28.	G. Zacharopoulos *et al.*, Predicting learning and achievement using GABA and glutamate concentrations in human development. *PLoS biology* **19**, e3001325 (2021).

29.	K. J. Friston, C. J. Price, Degeneracy and redundancy in cognitive anatomy. *Trends in cognitive sciences* **7**, 151-152 (2003).

30.	M. Amalric, S. Dehaene, Cortical circuits for mathematical knowledge: evidence for a major subdivision within the brain's semantic networks. *Philosophical Transactions of the Royal Society B: Biological Sciences* **373**, 20160515 (2018).

31.	M. Arsalidou, M. Pawliw-Levac, M. Sadeghi, J. Pascual-Leone, Brain areas associated with numbers and calculations in children: Meta-analyses of fMRI studies. *Developmental cognitive neuroscience* **30**, 239-250 (2018).

32.	Z. Hawes, H. M. Sokolowski, C. B. Ononye, D. Ansari, Neural underpinnings of numerical and spatial cognition: An fMRI meta-analysis of brain regions associated with symbolic number, arithmetic, and mental rotation. *Neuroscience & Biobehavioral Reviews* **103**, 316-336 (2019).

33.	E. Klein, A. Knops, The two-network framework of number processing: a step towards a better understanding of the neural origins of developmental dyscalculia. *Journal of Neural Transmission* **130**, 253-268 (2023).

34.	J. Siemann, F. Petermann, Evaluation of the Triple Code Model of numerical processing—Reviewing past neuroimaging and clinical findings. *Research in developmental disabilities* **72**, 106-117 (2018).

35.	O. Dakwar-Kawar *et al.*, Examining the effect of transcranial electrical stimulation and cognitive training on processing speed in pediatric attention deficit hyperactivity disorder: a pilot study. *Frontiers in human neuroscience* **16**, 791478 (2022).

36.	C. L. Gallen, S. Schaerlaeken, J. W. Younger, J. A. Anguera, A. Gazzaley, Contribution of sustained attention abilities to real-world academic skills in children. *Scientific reports* **13**, 2673 (2023).

37.	O. Van der Groen *et al.*, Using noise for the better: The effects of transcranial random noise stimulation on the brain and behavior. *Neuroscience & Biobehavioral Reviews* **138**, 104702 (2022).

38.	T. Popescu *et al.*, Transcranial random noise stimulation mitigates increased difficulty in an arithmetic learning task. *Neuropsychologia* **81**, 255-264 (2016).

39.	G. Edwards *et al.*, Prolonged neuromodulation of cortical networks following low-frequency rTMS and its potential for clinical interventions. *Frontiers in psychology* **10**, 529 (2019).

40.	J. Fan, B. D. McCandliss, T. Sommer, A. Raz, M. I. Posner, Testing the efficiency and independence of attentional networks. *Journal of cognitive neuroscience* **14**, 340-347 (2002).

41.	D. Wechsler, *Wechsler adult intelligence scale-revised (WAIS-R)* (Psychological corporation, San Antonio, TX, 1981).

42.	R. P. Kessels, M. J. Van Zandvoort, A. Postma, L. J. Kappelle, E. H. De Haan, The Corsi block-tapping task: standardization and normative data. *Applied neuropsychology* **7**, 252-258 (2000).

43.	M. Febo, T. C. Foster, Preclinical magnetic resonance imaging and spectroscopy studies of memory, aging, and cognitive decline. *Frontiers in aging neuroscience* **8**, 158 (2016).

44.	A. Lin *et al.*, Minimum reporting standards for in vivo magnetic resonance spectroscopy (MRSinMRS): experts' consensus recommendations. *NMR in Biomedicine* **34**, e4484 (2021).

45.	M. Declercq, E. Bellon, M. I. Sahan, W. Fias, B. De Smedt, Arithmetic learning in children: An fMRI training study. *Neuropsychologia* **169**, 108183 (2022).

46.	M. Delazer *et al.*, Number processing and basal ganglia dysfunction: a single case study. *Neuropsychologia* **42**, 1050-1062 (2004).

47.	K. Moeller, K. Willmes, E. Klein, A review on functional and structural brain connectivity in numerical cognition. *Frontiers in human neuroscience* **9**, 227 (2015).

48.	O. Matysiak, A. Kroemeke, A. Brzezicka, Working memory capacity as a predictor of cognitive training efficacy in the elderly population. *Frontiers in aging neuroscience* **11**, 126 (2019).

49.	D. Bissig, C. Lustig, Who benefits from memory training? *Psychological Science* **18**, 720-726 (2007).

50.	C. N. Bürki, C. Ludwig, C. Chicherio, A. de Ribaupierre, Individual differences in cognitive plasticity: an investigation of training curves in younger and older adults. *Psychological Research* **78**, 821-835 (2014).

51.	K. R. Kraemer, T. Enam, I. M. McDonough, Cognitive reserve moderates older adults’ memory errors in an autobiographical reality monitoring task. *Psychology & neuroscience* **12**, 247 (2019).

52.	M. Perc, The Matthew effect in empirical data. *Journal of The Royal Society Interface* **11**, 20140378 (2014).

53.	H. S. Scarborough, J. D. Parker, Matthew effects in children with learning disabilities: Development of reading, IQ, and psychosocial problems from grade 2 to grade 8. *Annals of Dyslexia* **53**, 47-71 (2003).

54.	P. L. Morgan, G. Farkas, J. Hibel, Matthew effects for whom? *Learning Disability Quarterly* **31**, 187-198 (2008).

55.	D. Duff, J. B. Tomblin, H. Catts, The influence of reading on vocabulary growth: A case for a Matthew effect. *Journal of Speech, Language, and Hearing Research* **58**, 853-864 (2015).

56.	Y. Kovas *et al.*, The genetic and environmental origins of learning abilities and disabilities in the early school years. *Monographs of the Society for research in Child Development*, i-156 (2007).

57.	K. Rimfeld *et al.*, The stability of educational achievement across school years is largely explained by genetic factors. *npj Science of Learning* **3**, 16 (2018).

58.	J. Liu *et al.*, Neuroanatomical, transcriptomic, and molecular correlates of math ability and their prognostic value for predicting learning outcomes. *Science Advances* **10**, eadk7220 (2024).

59.	N. Judd *et al.*, Cognitive and brain development is independently influenced by socioeconomic status and polygenic scores for educational attainment. *Proceedings of the National Academy of Sciences* **117**, 12411-12418 (2020).

60.	R. G. Pachella, in Human information processing: Tutorials in performance and cognition*,* B. H. Kantowitz, Ed. (Routledge, 2021), pp. 41-82.

61.	M. Rosenberg‐Lee *et al.*, Brain hyper‐connectivity and operation‐specific deficits during arithmetic problem solving in children with developmental dyscalculia. *Developmental science* **18**, 351-372 (2015).

62.	L. Michels, R. O’Gorman, K. Kucian, Functional hyperconnectivity vanishes in children with developmental dyscalculia after numerical intervention. *Developmental Cognitive Neuroscience* **30**, 291-303 (2018).

63.	K. Skagerlund *et al.*, Disentangling mathematics from executive functions by investigating unique functional connectivity patterns predictive of mathematics ability. *Journal of cognitive neuroscience* **31**, 560-573 (2019).

64.	R. A. Abreu‐Mendoza *et al.*, Parietal and hippocampal hyper‐connectivity is associated with low math achievement in adolescence–A preliminary study. *Developmental Science* **25**, e13187 (2022).

65.	D. Jolles *et al.*, Reconfiguration of parietal circuits with cognitive tutoring in elementary school children. *Cortex* **83**, 231-245 (2016).

66.	N. E. Van Bueren, S. H. Van Der Ven, K. Roelofs, R. Cohen Kadosh, E. H. Kroesbergen, Predicting math ability using working memory, number sense, and neurophysiology in children and adults. *Brain sciences* **12**, 550 (2022).

67.	T. Reed, R. Cohen Kadosh, Transcranial electrical stimulation (tES) mechanisms and its effects on cortical excitability and connectivity. *Journal of inherited metabolic disease* **41**, 1123-1130 (2018).

68.	C. J. Stagg, A. Antal, M. A. Nitsche, Physiology of transcranial direct current stimulation. *The journal of ECT* **34**, 144-152 (2018).

69.	M. A. Nitsche *et al.*, Modulating parameters of excitability during and after transcranial direct current stimulation of the human motor cortex. *The Journal of physiology* **568**, 291-303 (2005).
